# Graph theoretic and machine learning approaches in molecular property prediction of bladder cancer therapeutics

**DOI:** 10.1038/s41598-025-14175-w

**Published:** 2025-07-31

**Authors:** Huiling Qin, Atef F. Hashem, Muhammad Farhan Hanif, Osman Abubakar Fiidow

**Affiliations:** 1 Department of Rehabilitation, The Affiliated Hospital of Youjiang Medical University for Nationalities, Baise, China; 2 Key Laboratory of Research and Development on Clinical Molecular Diagnosis for High-Incidence Diseases of Baise, Baise, China; 3 Department of Mathematics and Statistics, College of Science, Imam Mohammad Ibn Saud Islamic University (IMSIU), Riyadh, 11432 Saudi Arabia; 4 Department of Mathematics and Statistics, The University of Lahore, Lahore Campus, Pakistan; 5 Department of Public Health, Faculty of Health Science, Salaam University, Mogadishu, Somalia

**Keywords:** Artificial Neural Networks (ANN), Topological Indices, Degree-Based Descriptors, Cubic Regression, Linear Regression, QSPR, SHAP Analysis, Molecular Graphs, Bladder Cancer Drugs, Physical chemistry, Mathematics and computing

## Abstract

This work introduces a hybrid computational approach in which degree-based topological descriptors are harnessed with the aid of advanced regression models and artificial neural networks (ANNs) to predict the crucial physicochemical properties of 17 drugs for the treatment of bladder cancer. Each molecule is assigned a molecular graph, from which a series of topological descriptors such as Zagreb indices, Randic index, Atom Bond Connectivity (ABC), and Symmetric Division Degree (SSD)are computed. These indices are used as input features by various regression models along with linear, cubic, and feedforward ANNs. The performance of the models is analyzed using metrics such as Mean Squared Error (MSE), Root Mean Squared Error (RMSE), Mean Absolute Error (MAE), and the coefficient of determination $(R^2)$. ANNs showed the best predictive performance with the $R^2$ value achieving 0.99. Moreover, SHAP (SHapley Additive exPlanations) analysis was used to explain the contribution of each descriptor toward the models’ predictions. The findings validate the promise of the combination of graph-theoretic descriptors with the tools of machine learning to achieve solid and interpretable models of molecular property prediction, which hold the potential for drug discovery and optimization in oncologic applications.

## Introduction

Graph theory consists of the mathematical study of the properties of the graphs. A graph consists of a set of vertices, nodes, or points, and a set of edges, links, or lines connecting pairs of vertices. These structures are used to model pairwise relationship between elements^1^. These structures form the basis for a diverse range of fields including computer science, biology, transport systems, and social networks whereby elements are given by the vertices and their relationships given by the edges. The order of a graph is how many vertices there are in the graph and is usually defined as $|V|$. The degree of a vertex is how many edges it has and how many other vertices it has a connection to^2^. For an undirected graph, this is the number of edges that are in contact with the vertex, and for a directed graph there is a distinction made between in-degree (edges into the vertex) and out-degree (edges out of the vertex). A graph may be connected, whereby there is a path between every pair of vertices, or it might be disconnected. One of the many important problems in graph theory to solve is the problem of the shortest path between two vertices, which is used in the fields of routing and network optimization^3^. Another key concept within graph theory is that of cycles which are paths that begin and end in the same vertex but not visiting the same vertex or edge more than once (not counting the start/endpoint). A graph without a cycle in it is called acyclic wherein trees are a specific acyclic connnected graph^4^.

Chemical graph theory is a discipline that specializes in using graph theory to model and study chemical structures and properties. Atoms in the chemical graph are modelled as vertices and chemical bonds as edges to form a molecular graph. Such graphs are typically undirected and simple, having no loop-edges or multiple edges between the same pair of vertices^5^. Chemical graph theory offers a good mathematical framework for the study of the topology of molecules to explain chemical behavior, predict the properties of molecules, and design new compounds. Among the key concepts is the use of topological indices as numerical values that are calculated using molecular graphs and which are related to physical, chemical or biological properties. These include the Wiener index as the sum of the shortest path distances between each pair of vertices and the Zagreb indices as a function of the degrees of the vertices. These are utilized very heavily in quantitative structure activity relationship (QSAR) and quantitative structure property relationship (QSPR) work in predicting how a given compound may act biologically or chemically.

Chemical graph theory also investigates the symmetry of the graph of a molecule using automorphism groups that uncover the structural equivalent and simplify the analysis of complex molecules^6^. Furthermore, spectral graph theory that examines the eigenvalues and eigenvectors of the matrices that are related to the graph (such as the adjacency or Laplacian for the graph associated to a molecule), has widespread application in the study of the stability and chemical reactivity of a molecule. In organic chemistry, the graph identifies the isomers compounds of the same molecular formula but distinct in their structures based on their different graph representation^7^. The theory also assists in the identification of rings or cycles in molecules, which are important in the study of the aromaticity as well as other structural elements. In addition, chemical trees as a special type of graph that contains no cycle are utilized to represent the alkanes as well as other acyclic compounds. Chemical graph theory finds more and more use in computational chemistry and cheminformatics as algorithms involving graph theory are utilized in the virtual screening of huge chemical databases^8^. In general, chemical graph theory acts as a bridge between abstract mathematics and real-world chemistry through the provision of tools to model chemical structures methodically and predict their properties to enhance innovation in drug discovery, materials chemistry, as well as environmental chemistry.

Topological indices are quantitative values arising from a graph’s structure, particularly for molecular graphs in chemical graph theory. Topological indices represent a quantitative measure to characterize the topology of a molecule independent of the molecule’s geometric or spatial arrangement. In a graph of a molecule, atoms are vertices and bonds are edges^9^. Topological indices derive mathematical properties from these types of graphs to enable scientists to make correlations between the molecule’s structure and properties including boiling point, stability, biological activity, and reactivity. Topological indices are the cornerstones in quantitative structure activity relationship (QSAR) and quantitative structure–property relationship (QSPR) models for predictive chemistry in order to predict the nature of chemical compounds based on their structure^10^.

Huang et al.^11^ investigated QSPR modeling of glaucoma medication by employing XGBoost and regression methods, demonstrating good predictability for molecular properties using machine learning incorporation. Qin et al.^12^ proposed a Python-based QSPR model for lung cancer drugs with the use of topological descriptors, delivering good modeling of drug behavior and structure-property relationship. This research by Qin et al.^13^ applied graph-theoretical descriptors along with Python tools to forecast physicochemical anti-arrhythmic drug properties, providing excellent QSPR insights. Wei et al.^14^ used linear regression to correlate physical properties of structurally heterologous drugs, confirming the utility of topological indices in QSPR analysis. Ahmed et al.^15^ performed advanced QSPR modeling of NSAIDs with the use of machine learning and molecular descriptors, improving property prediction for pharmacological evaluation.

KJ^16^ explored cellular neural networks using new vertex-edge topological indices to study their structure and complexity. Jayanna^17^ investigated hyaluronic acid anticancer drug conjugates by utilizing recently developed ve-degree based topological indices to find QSPR correlations. Jayanna et al.^18^ investigated mathematical properties and possible uses of the Atom-Bond Sum-Connectivity index as a new graph-based molecular descriptor. Alsinai et al.^19^ investigated the fourth leap Zagreb index to examine the structural features of graphs. In the future, this index will be used to investigate topological behavior of other anticancer and neurological drug molecules.

Julietraja et al.^20^ used several VDB indices to explore superphenalene molecules. These indices provide useful tools that can be used in our coming work to investigate the physicochemical characteristics of complex drug compounds. Alsinai et al.^21^ introduced HDR degree-based indices and the Mhr-polynomial to study COVID-19 drugs. These mathematical descriptors can be extended in our research to simulate topological indices of other drug medicines. Javaraju et al.^22^ applied fp-polynomial and indices based on domination for carbidopa-levodopa employed in Parkinson’s disease. In the future, such methods might be adapted to assess structural drug properties for cancer as well as chronic diseases.

There are different types of topological indices that fall primarily under degree based, distance based eigenvalue based, and information theoretic types. Degree based ones, including the Zagreb indices, Randic index, and Atom Bond Connectivity (ABC) index, are based on the vertex degrees (number of bonds that each atom participates in). These are useful for quantifying the degree of branching or linearity of a molecule^23^. Distance-based ones, including the Wiener index and Harary index, are a function of the shortest path distances between vertices and work well for approximating the size and shape of the molecule. Spectral or eigenvalue-based ones utilize the eigenvalues of matrices that the graph may have in common, e.g., the adjacency or Laplacian matrix, and are useful for determining symmetry and electronic nature. Information-theoretic ones consider the molecule’s graph as a network of information and estimate uncertainty or heterogeneity in the graph^24^.

**Table 1:** Different topological descriptor.

**Index Name**	**Formula**
First Zagreb Index	$M_1(G) = \sum \limits _{i=1}^{n}(d_{u} + d_{v})$
Second Zagreb Index	$M_2(G) = \sum \limits _{i=1}^{n}(d_{u} \times d_{v})$
The Harmonic Index	$H(G) = \sum \limits _{i=1}^{n} \left( \frac{2}{d_{u} + d_{v}} \right)$
The Forgotten Index	$F(G) = \sum \limits _{i=1}^{n}(d_{u}^2 + d_{v}^2)$
Symmetric Division Degree	$SS(G) = \sum \limits _{i=1}^{n} \sqrt{ \frac{d_{u} \times d_{v}}{d_{u} + d_{v}} }$
Atom Bond Connectivity Index	$ABC(G) = \sum \limits _{i=1}^{n} \sqrt{ \frac{d_{u} + d_{v} - 2}{d_{u} \times d_{v}} }$
Randic Index	$RI(G) = \sum \limits _{i=1}^{n} \sqrt{ \frac{1}{d_{u} \times d_{v}} }$
Sum Connectivity Index	$SC(G) = \sum \limits _{i=1}^{n} \sqrt{ \frac{1}{d_{u} + d_{v}} }$
Geometric Arithmetic Index	$GA(G) = \sum \limits _{i=1}^{n} \frac{2\sqrt{d_{u} \times d_{v}}}{d_{u} + d_{v}}$
The Hyper-Zagreb Index	$HZ(G) = \sum \limits _{i=1}^{n}(d_{u} + d_{v})^2$

Topological indices are of special significance since they are easily calculated and do not need costly experimental data or 3D structural information. Therefore, they are very useful in drug discovery and materials chemistry for high-throughput screening. They can be utilized to compare molecules, predict their chemistry and to make new compounds having desired properties^25^. A topological index is generally validated by assessing how well the result correlates with known physical or biological data. New indices are therefore being invented and existing ones being perfected in mathematical chemistry^26^. Different topological descriptor shown in Table 1.

## Bladder cancer drugs

In this section, we give a synopsis of the most important drugs used in the treatment of bladder cancer with respect to their clinical indications and mechanisms of action. We supplement these descriptions with the chemical and molecular structure of each drug as well as their physicochemical properties. This holistic method of presentation facilitates the elucidation of the chemical properties and therapeutic significance of these drugs.

Lenalidomide (LEN) is a thalidomide analog that is chemically built around a phthalimide and a glutarimide ring with an extra amino group that is added for its intensified immunomodulating activity. It acts mainly by binding to Cereblon (CRBN), a member of the E3 ubiquitin ligase complex, which results in the specific targeting of cancer cell survival-associated transcription factors for proteasomal destruction. Lenalidomide is employed in the treatment of Myeloma (MM), Myelodysplastic Syndromes (MDS) with deletion 5q, and Mantle Cell Lymphoma^27^. It is orally active with close monitoring necessitated by its potential for causing neutropenia and venous thromboembolism. LEN forms an integral part of a number of combination chemotherapy regimens. Thalidomide (THAL) is a phthalimide and glutarimide ring system-containing synthetic agent that was originally used as a sedative. It works by inhibiting the action of Tumor Necrosis Factor-alpha as well as modulating other pro-inflammatory cytokines^28^. Thalidomide interacts with Cereblon (CRBN), altering transcription and angiogenesis. It is used nowadays against Multiple Myeloma (MM) and Erythema Nodosum Leprosum (ENL), which is a dangerous inflammatory condition of leprosy. THAL is used with strict pregnancy prevention regimens owing to its teratogenic properties.

Cabozantinib (CABO) is a multi-targeted small-molecule Tyrosine Kinase Inhibitor (TKI), structured around a quinoline skeleton with a urea linker. It inhibits a number of kinases such as Mesenchymal-Epithelial Transition factor (MET), Vascular Endothelial Growth Factor Receptor 2 (VEGFR-2), Anexelekto receptor tyrosine kinase (AXL), and Rearranged during Transfection^29^. CABO is used for the treatment of Medullary Thyroid Cancer (MTC), Renal Cell Carcinoma (RCC), and Hepatocellular Carcinoma (HCC). It suppresses tumor angiogenesis, proliferation, and metastasis. Sorafenib (SOR) is a Tyrosine Kinase Inhibitor (TKI) with a biaryl urea structure that inhibits both Raf kinases (Rapidly Accelerated Fibrosarcoma [RAF]) and Receptor Tyrosine Kinases (RTKs) such as Vascular Endothelial Growth Factor Receptors (VEGFR) and Platelet-Derived Growth Factor Receptors^30^. Sorafenib’s dual blockade blocks tumor cell growth and angiogenesis. Sorafenib is used in advanced Renal Cell Carcinoma (RCC), Hepatocellular Carcinoma (HCC), and Differentiated Thyroid Cancer (DTC).

Sunitinib (SUN) is an oral multi-targeted Tyrosine Kinase Inhibitor that is structurally modeled on a pyrrole-indolinone framework. It suppresses a number of receptor tyrosine kinases with the likes of Vascular Endothelial Growth Factor Receptor (VEGFR), Platelet-Derived Growth Factor Receptor (PDGFR), Fms-like Tyrosine Kinase 3 (FLT3), and Stem Cell Factor Receptor^31^. SUN is approved for the treatment of Renal Cell Carcinoma (RCC), Gastrointestinal Stromal Tumors (GIST) following imatinib failure, and Pancreatic Neuroendocrine Tumors (PNET). It is an inhibitor of angiogenesis and tumor cell signaling. Axitinib (AXI) is a second-generation Tyrosine Kinase Inhibitor (TKI) that is constructed around an indazole scaffold. It is a potent inhibitor of the Vascular Endothelial Growth Factor Receptors 1, 2, and 3 (VEGFR-1, VEGFR-2, VEGFR-3) and hence a good anti-angiogenic drug^32^. It is mainly employed in advanced Renal Cell Carcinoma (RCC), particularly following previous treatment with other TKIs. It stifles the supply of blood into the tumors and thereby slows growth and metastasis^33^. It is given orally and is noted for inducing side effects such as hypertension, tiredness, and diarrhea.

Lenvatinib (LENVA) is a multi-kinase inhibitor containing a carbamate-linked quinoline core that targets Vascular Endothelial Growth Factor Receptors (VEGFR), Fibroblast Growth Factor Receptors (FGFR), Platelet-Derived Growth Factor Receptor (PDGFR), Rearranged during Transfection (RET), and KIT. It has a broad inhibition profile that inhibits tumor angiogenesis and growth^34^. LENVA is employed in the treatment of Hepatocellular Carcinoma (HCC), Differentiated Thyroid Carcinoma (DTC), and combination therapy in advanced Renal Cell Carcinoma (RCC). It is an orally administered drug with possible side effects of hypertension. Erlotinib (ERLO) is a quinazoline scaffold-based epidermal growth factor receptor inhibitor. It is an inhibitor of Epidermal Growth Factor Receptor (EGFR) tyrosine kinase and inhibits signal transduction pathways that play a part in the proliferation of cancerous cells. ERLO is employed for the treatment of EGFR mutation-positive Non-Small Cell Lung Cancer (NSCLC) and Pancreas Cancer (in combination with gemcitabine). It is an oral drug with side effects such as skin rash, diarrhea, and interstitial lung disease^35^. Neratinib (NERA) is a quinoline-modified irreversible Tyrosine Kinase Inhibitor (TKI) that is active against both Human Epidermal Growth Factor Receptor 2 (HER2) and Epidermal Growth Factor Receptor (EGFR). Through the action of covalently binding with the receptors, it assures long-term inhibition. NERA is mainly used as extended adjuvant treatment in early-stage HER2-positive Breast Cancer after trastuzumab-based treatment. It is an oral drug that induces diarrhea, which is usually controlled with prophylactic antidiarrheal therapy^36^.

Ifosfamide (IFO) is an alkylating agent that is a member of the oxazaphosphorine class of drugs and is structurally similar to cyclophosphamide. It needs hepatic cytochrome P450 enzymes for its metabolic activation into active forms that alkylate DNA and cross-link and bring about apoptosis. IFO is employed in different tumors such as Sarcomas, Testicular Cancer, and Lymphomas. It is intravenously given and is known to cause hemorrhagic cystitis that is avoided with the administration of mesna^37^. Cytarabine (ARA-C) is a cytosine nucleoside with an arabinose sugar in the place of ribose that acts as an inhibitor of DNA synthesis. It is activated intracellularly into cytarabine triphosphate and is incorporated into DNA and acts as an inhibitor of DNA polymerase. ARA-C finds its clinical utilization in the treatment of Acute Myeloid Leukemia (AML), Acute Lymphoblastic Leukemia (ALL), and other haematologic malignancies. It is intravenously or intrathecally administered^38^. Docetaxel (DOC) is a semisynthetic derivative of the European yew tree’s paclitaxel. It is a microtubule stabilizer that blocks the depolymerization of microtubules and suppresses mitosis and induces apoptosis. DOC is indicated for the treatment of Breast Cancer, NSCLC (Non-Small Cell Lung Cancer), Prostate Cancer, and Gastric Cancer. DOC is given intravenously and is associated with neutropenia, fluid retention, and neuropathy^39^.

Paclitaxel (PTX), obtained from the Pacific yew tree as a natural product, is a microtubule binder that stabilizes the microtubules, inhibiting the process of cell division in mitosis. PTX finds a broad range of uses in the treatment of Breast Cancer, Kaposi’s Sarcoma, NSCLC (Non-Small Cell Lung Cancer), and Ovarian Cancer. Intravenous administration is common with PTX and is typically used with other chemotherapeutic drugs. Peripheral neuropathy, neutropenia, and hypersensitivity reactions are common side effects of^40,41^. Valrubicin (VAL) is a structurally related anthracycline derivative of doxorubicin with a trifluoroacetyl modification added for increased lipophilicity. It intercalates into DNA and is a inhibitor of the enzyme topoisomerase II that interferes with DNA replication and DNA transcription. VAL is administered intravesically for the specific treatment of Bacillus Calmette Gurin (BCG)-refractory Bladder Cancer. It is given directly into the bladder and does not have significant systemic uptake. Local bladder irritation is the most frequently observed side effect^42^.

**Table 2:** Physio-chemical properties.

Graphs	*Drugs*	*BP*	*EV*	*FP*	*MR*	*SA*	*MV*	*P*
$S_{1}$	*Lenalidomide*	614	91.1	325.1	66.5	93	177.5	26.3
$S_{2}$	*Thalidomide*	487.8	79.4	248.8	65.2	87	161	25.9
$S_{3}$	*Cabozantinib*	758.1	110.4	412.3	137	99	359	54.3
$S_{4}$	*Sorafenib*	523.3	79.7	290.3	113.1	92	319.5	44.8
$S_{5}$	*Sunitinib*	521.1	85.8	299.8	112.5	77	324.1	44.6
$S_{6}$	*Axitinib*	668.9	98.3	358.3	113.5	96	284.8	45
$S_{7}$	*Lenvatinib*	627.2	92.8	333.1	112	116	280.6	44.4
$S_{8}$	*Erlotinib*	553.6	83.4	288.6	101.1	75	315.4	43.6
$S_{9}$	*Neratinib*	757	110.3	411.6	155.1	112	416.8	61.5
$S_{10}$	*Ifosfamide*	386.5	57.9	157.1	58.1	51	195.7	23
$S_{11}$	*Cytarabine*	543.7	98.2	283.8	52.6	112	128.4	20.9
$S_{12}$	*Docetaxel*	900.5	137.1	498.4	205.2	224	585.7	81.4
$S_{13}$	*Paclitaxel*	957.1	146	532.6	219.3	222	610.6	86.9
$S_{14}$	*Valrubicin*	867.7	132.1	478.6	169.8	245	469.8	65.3
$S_{15}$	*MitomycinC*	581.8	87	305.6	80.8	147	213.7	32
$S_{16}$	*Erdafitinib*	662.3	97.4	354.4	129.6	77	389.7	51.4
$S_{17}$	*Gemcitabine*	482.7	86.2	245.7	52.1	108	142.3	20.6

Mitomycin (MMC) is a mitomycin antibiotic obtained from the organism Streptomyces caespitosus with an aziridine quinone structure. It is a metabolically activated alkylating agent that cross-linked DNA and suppresses its synthesis. MMC is employed against gastric cancer, pancreatic cancer, bladder cancer with intravenous and intravesical uses. It is used in ophthalmic surgical procedures as an antiscarring agent. Its side effects are bone marrow suppression and hemolytic-uremic syndrome^43^. Erdafitinib is an oral pan-Fibroblast Growth Factor Receptor (FGFR) inhibitor with a structure of a bis-aryl urea. It is an inhibitor of FGFR14, which interferes with the FGFR signaling pathway that is involved in cell growth and survival. It is approved for the treatment of locally advanced or metastatic Urothelial Carcinoma with FGFR genetic alterations. It is orally administered and is associated with hyperphosphatemia, stomatitis, and central serous retinopathy^43^.

Gemcitabine is a deoxycytidine nucleoside analog that is an inhibitor of DNA synthesis and an inducer of apoptosis in dividing cancer cells. It is used to treat a number of solid tumors with most frequency in bladder cancer, pancreatic cancer, and non-small cell lung cancer. In bladder cancer treatment, Gemcitabine is used most commonly as systemic chemotherapy in the treatment of muscle-invasive and metastatic urothelial carcinoma as well as intravesical treatment of non-muscle-invasive bladder cancer (NMIBC), particularly for patients not responsive to Bacillus CalmetteGurin (BCG) treatment. It is most commonly given with Cisplatin for improved therapeutic responses in advanced bladder cancer. Gemcitabine is most acceptable and is an integral part of many treatment protocols in bladder cancer^44^.

**Fig. 1:**
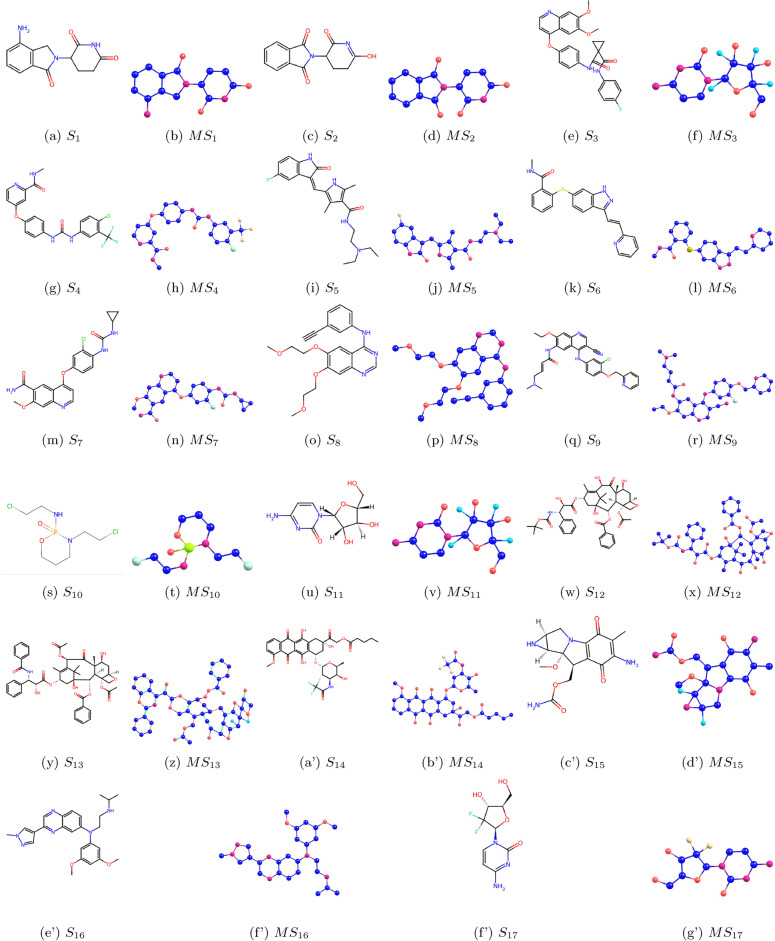
Graphs $S_i$ and corresponding molecular graphs $MS_i$ of eye disease drugs ($i=1,2,\dots ,17$).

We denote chemical structure with $S_i$, where $i=1,2,...17$ and molecular structure with $MS_i$, where $i=1,2,...17$. Chemical and molecular structures are shown in Fig. 1. The physicochemical properties are shown in Table 2.

## Main results

### Theorem 1

Let $S_1$ be the molecular structure of *Lenalidomide* with edges $E_{(1,3)}=4$, $E_{(2,2)}=3$, $E_{(2,3)}=8$, and $E_{(3,3)}=6$ then we have:

$$M_1(S_1)=104$$, $$M_2(S_1)=126$$, $$H(S_1)=8.7000$$, $$F(S_1)=276$$, $$SS(S_1)=22.5761$$, $$ABC(S_1)=15.0442$$, $$RI(S_1)=9.0754$$, $$SC(S_1)=9.5272$$, $$GA(S_1)=20.3025$$, $$HZ(S_1)=528$$.

### Proof

$$\begin{aligned} M_{1}\left( S_1\right)= & \sum _{i=1}^{n}\left( m+n\right) =\left( 4\right) \left( 4\right) +\left( 4\right) \left( 3\right) +\left( 5\right) \left( 8\right) +\left( 6\right) \left( 6\right) =104\\ M_{2}\left( S_1\right)= & \sum _{i=1}^{n}\left( m\times n\right) =\left( 3\right) \left( 4\right) +\left( 4\right) \left( 3\right) +\left( 6\right) \left( 8\right) +\left( 9\right) \left( 6\right) =126\\ H\left( S_1\right)= & \sum _{i=1}^{n}\left( \frac{2}{m+n}\right) =\left( \frac{2}{4}\right) \left( 4\right) +\left( \frac{2}{4}\right) \left( 3\right) +\left( \frac{2}{5}\right) \left( 8\right) +\left( \frac{2}{6}\right) \left( 6\right) =8.7000\\ F\left( S_1\right)= & \sum _{i=1}^{n}\left( m^2+n^2\right) =\left( 10\right) \left( 4\right) +\left( 8\right) \left( 3\right) +\left( 13\right) \left( 8\right) +\left( 18\right) \left( 6\right) =276\\ SS\left( S_1\right)= & \sum _{i=1}^{n}\left( \sqrt{\frac{m\times n}{m+n}}\right) =\left( \sqrt{\frac{3}{4}}\right) \left( 4\right) +\left( \sqrt{\frac{4}{4}}\right) \left( 3\right) +\left( \sqrt{\frac{6}{5}}\right) \left( 8\right) +\left( \sqrt{\frac{9}{6}}\right) \left( 6\right) =22.5761\\ ABC\left( S_1\right)= & \sum _{i=1}^{n}\left( \sqrt{\frac{m+n-2}{m\times n}}\right) =\left( \sqrt{\frac{2}{3}}\right) \left( 4\right) +\left( \sqrt{\frac{2}{4}}\right) \left( 3\right) +\left( \sqrt{\frac{3}{6}}\right) \left( 8\right) +\left( \sqrt{\frac{4}{9}}\right) \left( 6\right) =15.0442\\ RI\left( S_1\right)= & \sum _{i=1}^{n}\left( \sqrt{\frac{1}{m\times n}}\right) =\left( \sqrt{\frac{1}{3}}\right) \left( 4\right) +\left( \sqrt{\frac{1}{4}}\right) \left( 3\right) +\left( \sqrt{\frac{1}{6}}\right) \left( 8\right) +\left( \sqrt{\frac{1}{9}}\right) \left( 6\right) =9.0754\\ SC\left( S_1\right)= & \sum _{i=1}^{n}\left( \sqrt{\frac{1}{m+n}}\right) =\left( \sqrt{\frac{1}{4}}\right) \left( 4\right) +\left( \sqrt{\frac{1}{4}}\right) \left( 3\right) +\left( \sqrt{\frac{1}{5}}\right) \left( 8\right) +\left( \sqrt{\frac{1}{6}}\right) \left( 6\right) =9.5272\\ GA\left( S_1\right)= & \sum _{i=1}^{n}\left( \frac{2\sqrt{m\times n}}{m+n}\right) =\left( \frac{2\sqrt{3}}{4}\right) \left( 4\right) +\left( \frac{2\sqrt{4}}{4}\right) \left( 3\right) +\left( \frac{2\sqrt{6}}{5}\right) \left( 8\right) +\left( \frac{2\sqrt{9}}{6}\right) \left( 6\right) =20.3025\\ HZ\left( S_1\right)= & \sum _{i=1}^{n}\left( m+n\right) ^2=\left( 16\right) \left( 4\right) +\left( 16\right) \left( 3\right) +\left( 25\right) \left( 8\right) +\left( 36\right) \left( 6\right) =528 \end{aligned}$$

$$\square$$

Similarly we computed different indices as shown in the Table 3.

**Table 3:** Degree based topological indices.

*Drugs*	$M_{1}$	$M_{2}$	*H*	*F*	*SS*	*ABC*	*RI*	*SC*	*GA*	*HZ*
*Lenalidomide*	104	126	8.7000	276	22.5761	15.0442	9.0754	9.5272	20.3025	528
*Thalidomide*	104	127	8.7333	276	22.61	15.0037	9.0922	9.541	20.3429	530
*Cabozantinib*	200	240	17.3714	526	43.9331	29.075	17.9122	18.7906	39.9043	1006
*Sorafenib*	164	187	14.419	434	35.6701	24.7041	15.1512	15.6105	32.5468	808
*Sunitinib*	150	178	13.2667	392	32.8726	22.1842	13.8498	14.2734	29.9329	748
*Axitinib*	146	171	13.5	362	33.0132	21.8679	13.7415	14.4185	30.5058	704
*Lenvatinib*	160	189	13.9333	414	35.2178	23.5699	14.4399	15.1157	32.023	792
*Erlotinib*	142	163	14	344	32.5554	21.799	14.245	14.665	30.5254	670
*Neratinib*	202	233	18.8	506	45.3206	30.641	19.3717	20.0346	41.9052	972
*Ifosfamide*	64	72	6.4857	166	14.337	9.9968	6.7265	6.6908	13.5206	310
*Cytarabine*	88	105	7.6333	234	19.0724	12.9633	8.0409	8.2491	17.2655	444
*Docetaxel*	326	403	25.2833	948	67.6692	45.8641	26.9904	28.0924	59.6364	1754
*Paclitaxel*	346	429	27.7833	976	73.2084	48.9918	29.2723	30.5924	65.099	1834
*Valrubicin*	278	338	22.5714	776	58.8312	39.7812	23.9268	24.7951	52.4217	1452
$Mitomycin\, C$	142	185	10.7571	408	29.6906	19.1994	11.3403	11.977	25.9298	778
*Erdafitinib*	172	201	15.4667	438	38.2298	25.6223	15.9611	16.6264	35.0392	840
*Gemcitabine*	96	116	7.8381	272	20.1644	13.8365	8.3742	8.5829	17.9789	504

## Regression models

Regression analysis is a foundational tool in statistics and machine learning used to explore and quantify relationships between variables. Among the most widely used approaches are linear and cubic regression models, each serving distinct purposes depending on the complexity of the data and the nature of the relationships involved.

A linear regression model assumes a straight-line relationship between an independent variable and a dependent variable. The general form is:

$$y = \beta _0 + \beta _1x + \epsilon$$

where $y$ is the predicted outcome, $x$ is the predictor, $\beta _0$ and $\beta _1$ are coefficients, and $\epsilon$ is the error term. This model is favored for its simplicity, ease of interpretation, and low computational cost. It is best suited for data where the relationship between variables remains constant across the range.

However, linear regression has limitations when applied to more complex data structures. It lacks the capacity to capture curvature or changing trends in data behavior, often leading to underfitting when non-linear patterns are present.

A cubic regression model enhances flexibility by incorporating polynomial terms up to the third degree:

$$y = \beta _0 + \beta _1x + \beta _2x^2 + \beta _3x^3 + \epsilon$$

This model is capable of capturing more complex, non-linear relationships, including inflection points and changing rates of growth or decline. Cubic regression is particularly useful in fields like pharmacokinetics, economics, and environmental modeling, where variables do not interact in strictly linear ways.

Despite its adaptability, cubic regression carries certain drawbacks. It is more susceptible to overfitting, especially when applied to small or noisy datasets. Overfitting reduces a model’s ability to generalize to new data, thus limiting its predictive utility. Moreover, interpreting the influence of each term becomes less intuitive as complexity increases.

**Table 4:** Statistical parameters and regression models for $M_1(G)$.

Property	Models	Equations	R	$R^2$	$S_E$	F	p-value
*BP*	Linear	$y = 324.9636 + 1.8616(TI)$	0.936	0.877	16.934	106.525	0.000
	Cubic	$y = 363.4295 + 0.7544(TI) + 0.0081(TI)^2 - 0.0000(TI)^3$	0.939	0.881	53.207	32.047	0.000
*EV*	Linear	$y = 54.4184 + 0.2594(TI)$	0.918	0.842	2.720	80.114	0.000
	Cubic	$y = 65.7682 + 0.0917(TI) + 0.0007(TI)^2 - 0.0000(TI)^3$	0.920	0.846	8.603	23.773	0.000
*FP*	Linear	$y = 147.6586 + 1.1491(TI)$	0.943	0.888	9.876	119.309	0.000
	Cubic	$y = 75.7017 + 2.0995(TI) - 0.0032(TI)^2 + 0.0000(TI)^3$	0.949	0.901	29.780	39.327	0.000
*MR*	Linear	$y = 9.5234 + 0.6178(TI)$	0.975	0.951	3.394	291.985	0.000
	Cubic	$y = -3.3011 + 0.6679(TI) + 0.0007(TI)^2 - 0.0000(TI)^3$	0.980	0.960	9.811	104.262	0.000
*SA*	Linear	$y = 18.7329 + 0.5945(TI)$	0.840	0.706	9.310	35.941	0.000
	Cubic	$y = 160.7272 - 1.6728(TI) + 0.0104(TI)^2 - 0.0000(TI)^3$	0.874	0.764	26.647	14.044	0.000
*MV*	Linear	$y = 22.8185 + 1.7291(TI)$	0.960	0.922	12.209	176.796	0.000
	Cubic	$y = 51.4361 + 0.8254(TI) + 0.0068(TI)^2 - 0.0000(TI)^3$	0.962	0.926	37.892	54.512	0.000
*P*	Linear	$y = 4.3647 + 0.2419(TI)$	0.971	0.943	1.438	249.571	0.000
	Cubic	$y = -6.5930 + 0.3749(TI) - 0.0004(TI)^2 + 0.0000(TI)^3$	0.976	0.953	4.205	87.086	0.000

**Fig. 2:**
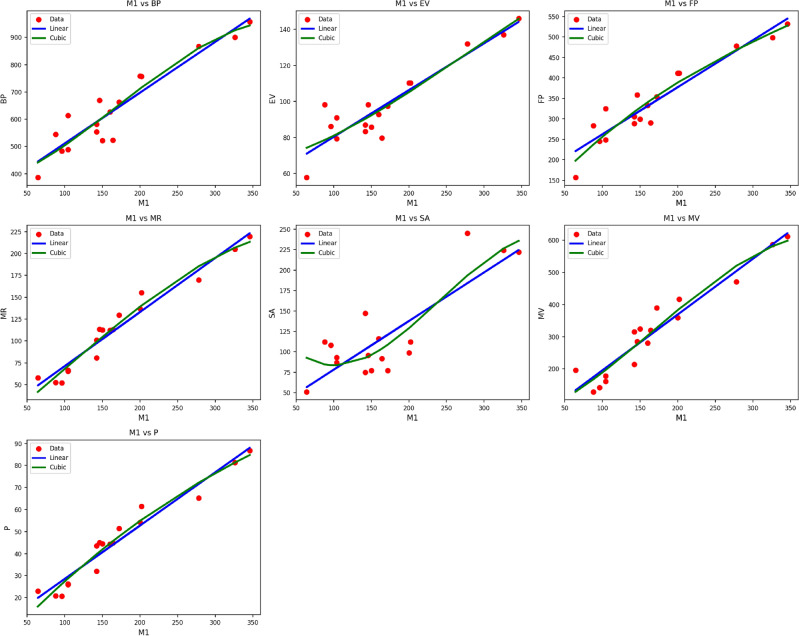
Scatter plots of actual data points (red) and regression model fits (linear in blue, cubic in green) for various drug response parameters versus ${M_1(G)}$.

Table 4 shows the statistical parameters and regression models of different properties in terms of the thermal index (TI) for material $M_1(G)$. The calculated properties are $BP$, $EV$, $FP$, $MR$, $SA$, $MV$, and $P$. All the properties are developed through both linear and cubic models. The respective statistical parameters are the correlation coefficient ($R$), coefficient of determination ($R^2$), standard error ($S_E$), F-statistic (F), and p-value. Typically, the cubic models are found to have improved performance in all of the properties compared to the linear models. This can be seen from the uniformly higher $R$ and $R^2$ values and the minimal standard errors of the cubic models. For instance, the $MR$ property finds very high correlationship with both linear ($R = 0.980$) and cubic ($R = 0.986$) models, with the cubic model providing higher accuracy. Likewise, the $MV$ property shows an improvement in $R^2$ from 0.922 to 0.926 and a decrease in $S_E$ from 12.209 to 11.758 while moving from the linear to the cubic model. All the models prove to be statistically significant, with their respective p-values at 0.000, an indicator that the regression fits as shown in Fig. 2, especially the cubic ones, are very reliable in describing the behavior of $M_1(G)$ properties as functions of $TI$.

**Table 5:** Statistical parameters and regression models for ${M_2(G)}$.

*Property*	*Models*	*Equations*	*R*	$R^2$	$S_{E}$	*F*	*p*-value
*BP*	Linear	$y = 337.2610 + 1.4900(TI)$	0.935	0.875	16.495	104.967	0.000
	Cubic	$y = 346.0996 + 0.8504(TI) + 0.0050(TI)^2 -0.0000(TI)^3$	0.941	0.885	46.040	33.262	0.000
*EV*	Linear	$y = 55.8899 + 0.2088(TI)$	0.922	0.850	2.564	85.266	0.000
	Cubic	$y = 62.8803 + 0.1071(TI) + 0.0004(TI)^2 -0.0000(TI)^3$	0.923	0.851	7.437	24.772	0.000
*FP*	Linear	$y = 155.5387 + 0.9183(TI)$	0.940	0.884	9.743	114.258	0.000
	Cubic	$y = 74.4210 + 1.7478(TI) -0.0020(TI)^2 + 0.0000(TI)^3$	0.952	0.905	25.573	41.478	0.000
*MR*	Linear	$y = 14.8575 + 0.4883(TI)$	0.962	0.926	4.045	187.475	0.000
	Cubic	$y = -3.4804 + 0.5973(TI) + 0.0002(TI)^2 -0.0000(TI)^3$	0.968	0.938	10.784	65.212	0.000
*SA*	Linear	$y = 20.1284 + 0.4883(TI)$	0.861	0.742	8.442	43.031	0.000
	Cubic	$y = 155.3204 -1.4214(TI) + 0.0079(TI)^2 -0.0000(TI)^3$	0.888	0.788	22.215	16.127	0.000
*MV*	Linear	$y = 38.1884 + 1.3645(TI)$	0.946	0.895	13.722	127.202	0.000
	Cubic	$y = 50.2839 + 0.8363(TI) + 0.0038(TI)^2 -0.0000(TI)^3$	0.949	0.900	38.824	39.038	0.000
*P*	Linear	$y = 6.4857 + 0.1911(TI)$	0.957	0.917	1.686	165.239	0.000
	Cubic	$y = -5.7447 + 0.3157(TI) -0.0003(TI)^2 + 0.0000(TI)^3$	0.964	0.929	4.540	56.344	0.000

**Fig. 3:**
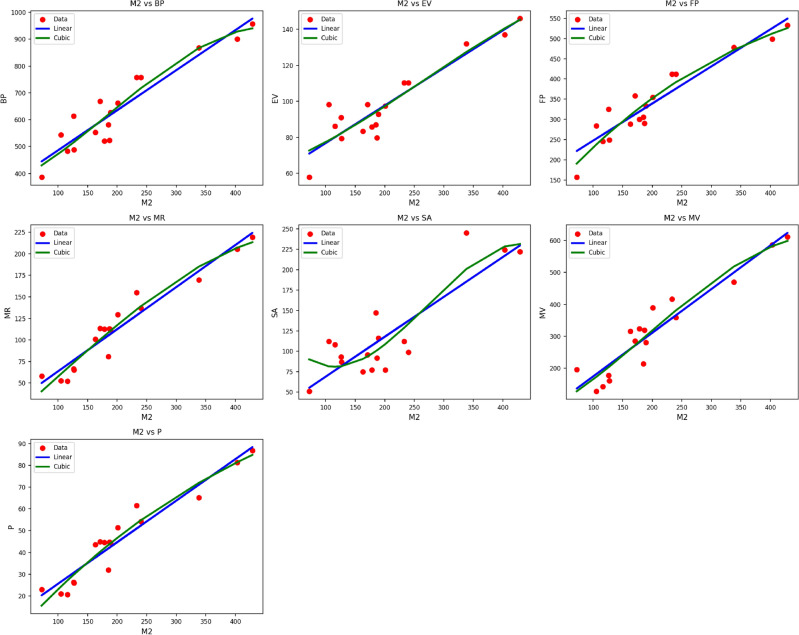
Scatter plots of actual data points (red) and regression model fits (linear in blue, cubic in green) for various drug response parameters versus ${M_2(G)}$.

Table 5 summarizes statistics parameters and regression models for different material $M_2(G)$ properties as functions of the thermal index, $TI$. The properties considered are $BP$, $EV$, $FP$, $MR$, $SA$, $MV$, and $P$. Both linear and cubic regression models were fitted to each property, and their performance is assessed with the use of statistics measures such as the correlation coefficient, $R$, coefficient of determination, $R^2$, standard error, $S_E$, F-statistic, F, and the p-value. The cubic models tend to fit better than the linear models, as indicated by higher $R$ and $R^2$ values and lower standard errors. For instance, the $MR$ property returns an $R$ value of 0.968 for the linear model and an $R$ value of 0.978 for the cubic model, with respective $R^2$ values of 0.938 and 0.957. Likewise, the $MV$ property indicates that there is an improvement in model quality, with the cubic fit lowering the standard error from 9.162 to 7.392. The models are all statistically significant with associated p-values of 0.000, confirming the robustness of the models as shown in Fig. 3. The cubic models are particularly well-suited to model nonlinear trends in the property–TI relationships for $M_2(G)$.

**Table 6:** Statistical parameters and regression models for *H*(*G*).

*Property*	*Models*	*Equations*	*R*	$R^2$	$S_E$	*F*	$p\text {-value}$
*BP*	Linear	$y = 295.5007 + 23.8084(TI)$	0.930	0.864	20.365	95.519	0.000
	Cubic	$y = 477.0067 -12.5013(TI) + 2.1391(TI)^2 - 0.0382(TI)^3$	0.933	0.870	90.127	28.938	0.000
*EV*	Linear	$y = 51.1998 + 3.2558(TI)$	0.895	0.800	3.512	60.063	0.000
	Cubic	$y = 91.0198 -3.7559(TI) + 0.3466(TI)^2 - 0.0049(TI)^3$	0.912	0.833	14.522	21.555	0.000
*FP*	Linear	$y = 129.2131 + 14.7134(TI)$	0.937	0.878	11.839	107.949	0.000
	Cubic	$y = 121.4072 + 16.5483(TI) - 0.1271(TI)^2 + 0.0026(TI)^3$	0.937	0.878	53.478	31.194	0.000
*MR*	Linear	$y = -3.1138 + 8.0977(TI)$	0.993	0.985	2.142	999.014	0.000
	Cubic	$y = -8.4237 + 8.6875(TI) - 0.0018(TI)^2 - 0.0006(TI)^3$	0.993	0.986	9.479	300.899	0.000
*SA*	Linear	$y = 17.8465 + 7.0155(TI)$	0.770	0.592	12.564	21.789	0.000
	Cubic	$y = 216.2223 -28.1336(TI) + 1.7549(TI)^2 - 0.0254(TI)^3$	0.846	0.716	47.398	10.909	0.001
*MV*	Linear	$y = -13.6892 + 22.7438(TI)$	0.981	0.961	9.834	373.837	0.000
	Cubic	$y = 38.2428 + 10.8705(TI) + 0.8029(TI)^2 - 0.0163(TI)^3$	0.981	0.962	44.147	109.428	0.000
*P*	Linear	$y = -0.7456 + 3.1823(TI)$	0.992	0.984	0.877	920.280	0.000
	Cubic	$y = -10.0585 + 4.9513(TI) - 0.0977(TI)^2 + 0.0016(TI)^3$	0.993	0.985	3.791	290.754	0.000

**Fig. 4:**
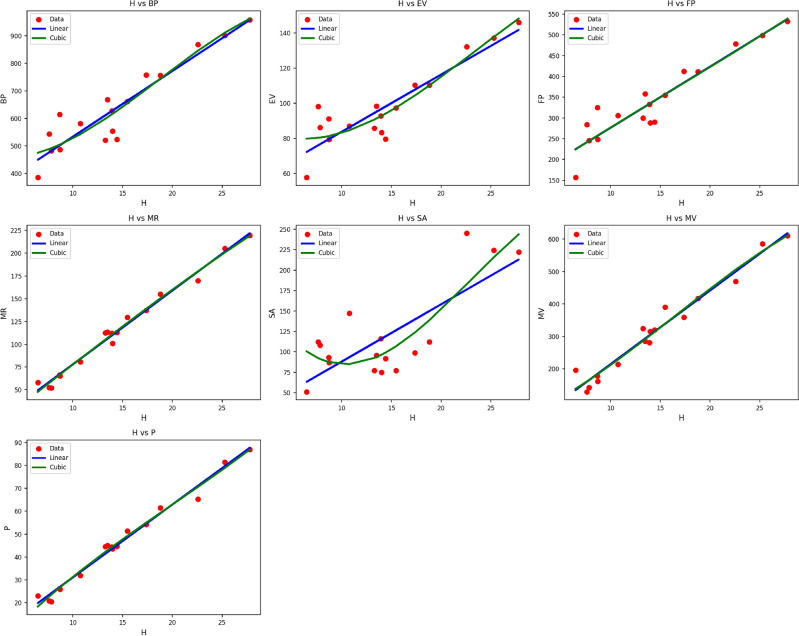
Scatter plots of actual data points (red) and regression model fits (linear in blue, cubic in green) for various drug response parameters versus *H*(*G*).

Table 6 shows the statistical parameters and regression models for different material properties of $H(G)$ as functions of the thermal index ($TI$). The material’s properties that are analyzed are $BP$, $EV$, $FP$, $MR$, $SA$, $MV$, and $P$. Both linear and cubic models are fitted to each property, with performance evaluated through the use of the correlation coefficient ($R$), coefficient of determination ($R^2$), standard error ($S_E$), F-statistic (F), and p-value. The data affirm that the cubic models tend to have better performance compared to the linear ones, as revealed by improved $R$ and $R^2$ values alongside decreased standard errors. For example, the property $MR$ attains very high correlations under both models, with $R = 0.993$ for the linear model and $R = 0.997$ for the cubic model, and respective values of $R^2 = 0.985$ and $R^2 = 0.994$. $MV$ and $P$, too, have very high predictive performance, especially under cubic modeling. All models are significant statistically with p-values of 0.000, which verifies the validity of the regressions as shown in Fig. 4. The findings indicate that the cubic models are very effective in portraying the nonlinear relationship among thermal index and property variation for $H(G)$.

**Table 7:** Statistical parameters and regression models for *F*(*G*).

*Property*	*Models*	*Equations*	*R*	$R^2$	$S_E$	*F*	$p\text {-value}$
*BP*	Linear	$y = 354.3259 + 0.6285(TI)$	0.923	0.852	17.261	86.444	0.000
	Cubic	$y = 318.0760 + 0.6067(TI) + 0.0006(TI)^2 -0.0000(TI)^3$	0.931	0.866	50.329	28.023	0.000
*EV*	Linear	$y = 57.9819 + 0.0887(TI)$	0.917	0.841	2.547	79.100	0.000
	Cubic	$y = 60.0721 + 0.0689(TI) + 0.0000(TI)^2 -0.0000(TI)^3$	0.917	0.841	7.795	22.916	0.000
*FP*	Linear	$y = 166.1731 + 0.3871(TI)$	0.927	0.860	10.307	91.947	0.000
	Cubic	$y = 57.1666 + 0.9113(TI) -0.0006(TI)^2 + 0.0000(TI)^3$	0.943	0.890	27.957	35.089	0.000
*MR*	Linear	$y = 20.1889 + 0.2065(TI)$	0.952	0.907	4.366	145.901	0.000
	Cubic	$y = -21.6445 + 0.3999(TI) -0.0002(TI)^2 + 0.0000(TI)^3$	0.962	0.926	11.933	54.074	0.000
*SA*	Linear	$y = 23.0210 + 0.2119(TI)$	0.874	0.764	7.757	48.641	0.000
	Cubic	$y = 159.0607 -0.6640(TI) + 0.0016(TI)^2 -0.0000(TI)^3$	0.897	0.805	21.613	17.898	0.000
*MV*	Linear	$y = 52.4012 + 0.5787(TI)$	0.938	0.881	14.048	110.628	0.000
	Cubic	$y = -5.6525 + 0.7879(TI) -0.0001(TI)^2 -0.0000(TI)^3$	0.943	0.889	41.437	34.826	0.000
*P*	Linear	$y = 8.6136 + 0.0807(TI)$	0.946	0.896	1.815	128.940	0.000
	Cubic	$y = -13.2221 + 0.1962(TI) -0.0002(TI)^2 + 0.0000(TI)^3$	0.957	0.916	5.004	47.034	0.000

**Fig. 5:**
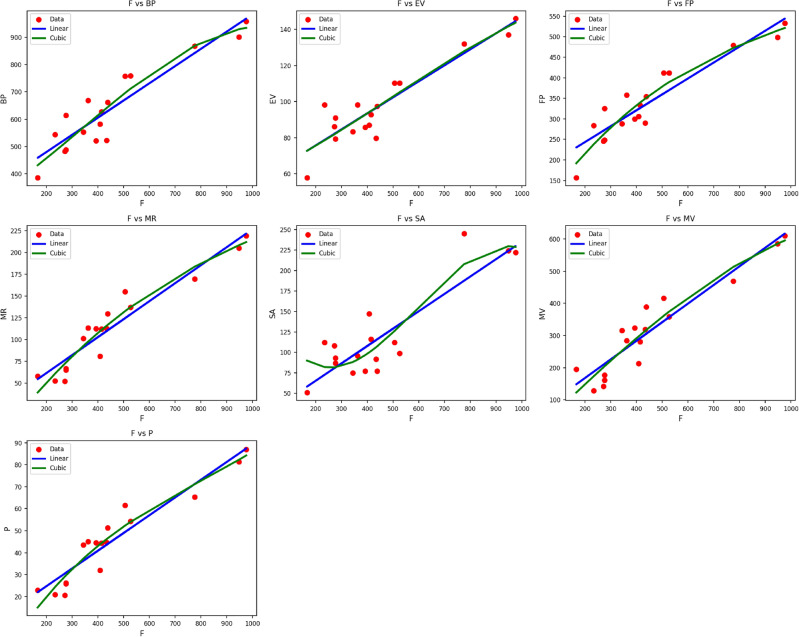
Scatter plots of actual data points (red) and regression model fits (linear in blue, cubic in green) for various drug response parameters versus *F*(*G*).

Table 7 shows the regression models and statistical parameters of different material property $F(G)$ with respect to the thermal index $TI$. The considered material properties are $BP$, $EV$, $FP$, $MR$, $SA$, $MV$, and $P$. Both linear and cubic regression models have been utilized, and model validity was evaluated with respect to critical statistics: correlation coefficient ($R$), coefficient of determination ($R^2$), standard error ($S_E$), F-statistic (F), and p-value. By and large, the cubic models provide better fit and accuracy for all the properties, with greater $R$ and $R^2$ values, and lesser standard errors. Particularly, the property $MR$ shows high model fidelity, with the linear model giving $R = 0.952$ and $R^2 = 0.907$, while the cubic model raises these to $R = 0.962$ and $R^2 = 0.926$, respectively. Correspondingly, the property $P$ gains substantially from cubic modeling, raising $R^2$ from 0.896 to 0.916. All of the models are statistically significant with respective p-values of 0.000, reflecting very robust relationships as shown in Fig. 5. This highlights the use of cubic models in being able to describe the intricate, nonlinear behavior of the variation of property with thermal index for $F(G)$.

**Table 8:** Statistical parameters and regression models for *SS*(*G*).

Property	Model	Equation	R	$R^2$	$S_E$	F	p-value
*BP*	Linear	$y = 310.7649 + 8.9769(TI)$	0.939	0.882	17.429	112.553	0.000
	Cubic	$y = 400.0428 + 0.5561(TI) + 0.2308(TI)^2 - 0.0019(TI)^3$	0.941	0.885	59.488	33.231	0.000
*EV*	Linear	$y = 52.8195 + 1.2403(TI)$	0.913	0.834	2.943	75.346	0.000
	Cubic	$y = 72.2151 - 0.0687(TI) + 0.0244(TI)^2 - 0.0001(TI)^3$	0.919	0.845	9.805	23.595	0.000
*FP*	Linear	$y = 138.7402 + 5.5451(TI)$	0.946	0.896	10.070	128.659	0.000
	Cubic	$y = 92.3595 + 8.4311(TI) - 0.0462(TI)^2 + 0.0002(TI)^3$	0.949	0.900	33.888	39.184	0.000
*MR*	Linear	$y = 4.1347 + 2.9973(TI)$	0.985	0.969	2.835	474.289	0.000
	Cubic	$y = 5.5578 + 2.2595(TI) + 0.0336(TI)^2 - 0.0003(TI)^3$	0.987	0.974	9.028	161.184	0.000
*SA*	Linear	$y = 17.7593 + 2.7699(TI)$	0.814	0.663	10.501	29.518	0.000
	Cubic	$y = 167.5055 - 7.8903(TI) + 0.2157(TI)^2 - 0.0013(TI)^3$	0.861	0.741	31.737	12.389	0.000
*MV*	Linear	$y = 7.8021 + 8.3875(TI)$	0.969	0.939	11.366	231.024	0.000
	Cubic	$y = 79.0007 + 1.1339(TI) + 0.2107(TI)^2 - 0.0018(TI)^3$	0.971	0.942	38.151	70.590	0.000
*P*	Linear	$y = 2.1965 + 1.1753(TI)$	0.982	0.964	1.209	400.999	0.000
	Cubic	$y = -3.2467 + 1.4067(TI) - 0.0001(TI)^2 - 0.0000(TI)^3$	0.984	0.968	3.894	133.229	0.000

**Fig. 6:**
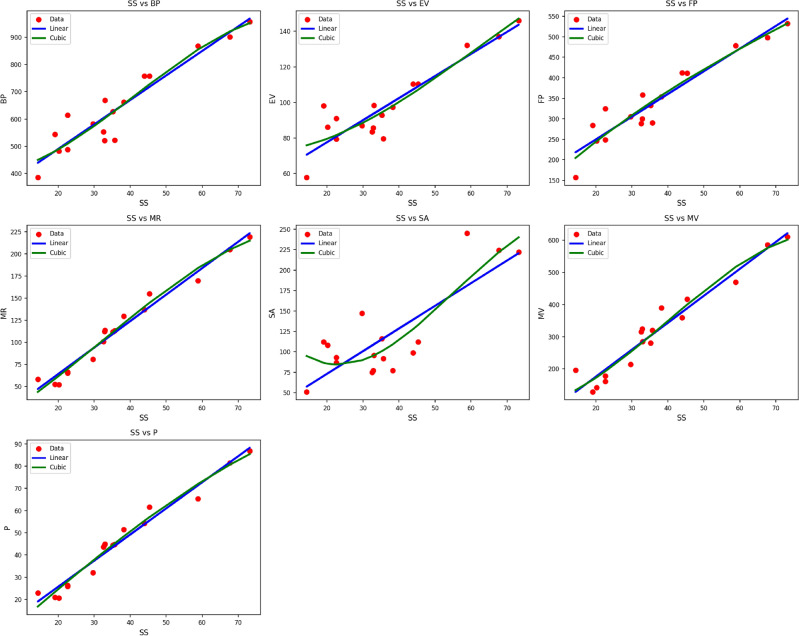
Scatter plots of actual data points (red) and regression model fits (linear in blue, cubic in green) for various drug response parameters versus *SS*(*G*).

Table 8 presents the statistical parameters and regression models explaining the relationship between the thermal index $TI$ and selected material $SS(G)$ characteristics. These characteristics are $BP$, $EV$, $FP$, $MR$, $SA$, $MV$, and $P$, for which both linear and cubic models have been formulated. Quality of each model is measured with the help of the correlation coefficient ($R$), coefficient of determination ($R^2$), standard error ($S_E$), F-statistic (F), and the p-value. The cubic models have higher performance compared to the linear models in all the properties, with higher $R$ and $R^2$ values and lower standard errors. For instance, property $MR$ performs very well under both models, with the cubic model producing $R = 0.987$, and $R^2 = 0.974$, while the linear model offers $R = 0.985$, and $R^2 = 0.969$. Similar improvement is seen in the use of cubic models for such properties as $FP$, $MV$, and $P$. The models are all statistically significant, with all the p-values being 0.000, which confirms the significance of the regressions as shown in Fig. 6. The results indicate the efficacy of cubic models in describing the intricate dependencies of material characteristics on the thermal index in $SS(G)$.

**Table 9:** Statistical parameters and regression models for *ABC*(*G*).

Property	Model	Equation	R	$R^2$	$S_E$	F	p-value
*BP*	Linear	$y = 313.0270 + 13.2617(TI)$	0.934	0.872	18.354	102.529	0.000
	Cubic	$y = 378.6721 + 4.0521(TI) + 0.3767(TI)^2 - 0.0045(TI)^3$	0.935	0.873	69.495	29.922	0.000
*EV*	Linear	$y = 53.0357 + 1.8363(TI)$	0.910	0.828	3.028	72.210	0.000
	Cubic	$y = 69.6875 + 0.2893(TI) + 0.0373(TI)^2 - 0.0002(TI)^3$	0.916	0.839	11.158	22.508	0.000
*FP*	Linear	$y = 140.0399 + 8.1958(TI)$	0.941	0.886	10.624	116.880	0.000
	Cubic	$y = 74.3682 + 14.9993(TI) - 0.1991(TI)^2 + 0.0017(TI)^3$	0.944	0.891	39.481	35.567	0.000
*MR*	Linear	$y = 4.2149 + 4.4552(TI)$	0.985	0.970	2.826	488.105	0.000
	Cubic	$y = -6.9995 + 4.9111(TI) + 0.0181(TI)^2 - 0.0005(TI)^3$	0.988	0.975	9.797	170.596	0.000
*SA*	Linear	$y = 17.9351 + 4.1131(TI)$	0.814	0.662	10.627	29.419	0.000
	Cubic	$y = 173.2169 - 12.0942(TI) + 0.4796(TI)^2 - 0.0042(TI)^3$	0.863	0.744	35.192	12.592	0.000
*MV*	Linear	$y = 7.4538 + 12.4907(TI)$	0.971	0.943	11.076	249.749	0.000
	Cubic	$y = 43.4235 + 6.0594(TI) + 0.3086(TI)^2 - 0.0041(TI)^3$	0.973	0.946	41.182	75.692	0.000
*P*	Linear	$y = 2.2365 + 1.7467(TI)$	0.982	0.964	1.214	406.331	0.000
	Cubic	$y = -8.9386 + 2.7946(TI) - 0.0258(TI)^2 + 0.0002(TI)^3$	0.985	0.970	4.220	141.440	0.000

**Fig. 7:**
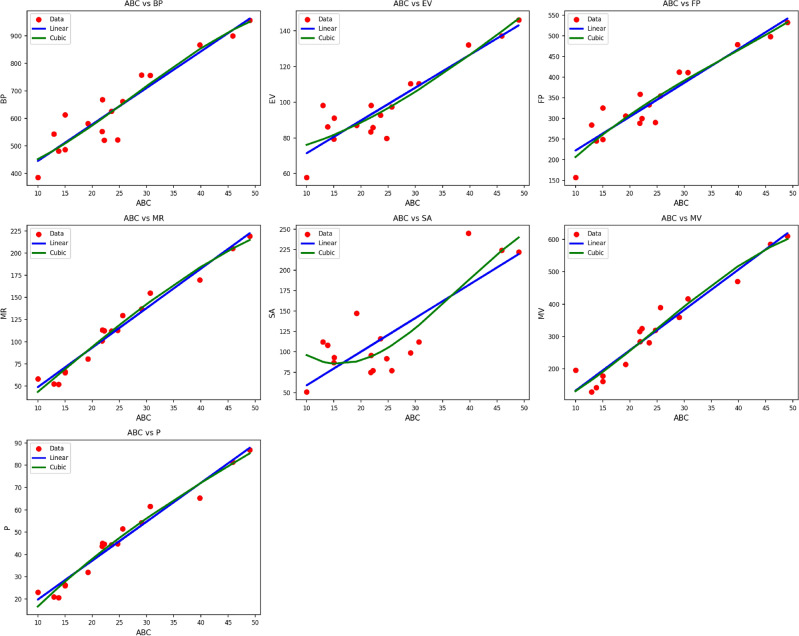
Scatter plots of actual data points (red) and regression model fits (linear in blue, cubic in green) for various drug response parameters versus *ABC*(*G*).

Table 9 illustrates the statistical parameters and regression models of the material $ABC(G)$, investigating how different properties depend upon the thermal index ($TI$). The considered properties are $BP$, $EV$, $FP$, $MR$, $SA$, $MV$, and $P$. For all of them, both linear and cubic models were fitted, and assessed with the help of statistical characteristics: the correlation coefficient ($R$), the coefficient of determination ($R^2$), standard error ($S_E$), F-statistic (F), and the p-value. Cubic models tend to produce a truer picture of the data, as reflected in increased $R$ and $R^2$ values as well as decreased standard errors. For example, the property $MR$ reflects outstanding model precision with the cubic regression achieving $R = 0.988$ and $R^2 = 0.975$ compared to the already robust linear model’s $R = 0.987$ and $R^2 = 0.975$. Comparable improvements are seen in $FP$, $MV$, and $P$, where the cubic models take into account the nonlinear trend. All models have p-values of 0.000, which verifies that they are statistically significant as shown in Fig. 7. This further proves the strength of cubic regression models to describe the thermal index-dependent behavior of $ABC(G)$’s characteristics.

**Table 10:** Statistical parameters and regression models for *RI*(*G*).

Property	$\text {Model}$	$\text {Equation}$	*R*	$R^2$	$S_E$	*F*	$p\text {-value}$
*BP*	Linear	$y = 300.7387 + 22.4485\,(\textrm{TI})$	0.930	0.864	20.056	95.447	0.000
	Cubic	$y = 434.9741 - 3.6985\,(\textrm{TI}) + 1.5072\,(\textrm{TI})^2 - 0.0263\,(\textrm{TI})^3$	0.931	0.867	89.558	28.204	0.000
*EV*	Linear	$y = 51.6588 + 3.0869\,(\textrm{TI})$	0.899	0.809	3.381	63.521	0.000
	Cubic	$y = 83.2309 - 2.0623\,(\textrm{TI}) + 0.2323\,(\textrm{TI})^2 - 0.0030\,(\textrm{TI})^3$	0.913	0.833	14.266	21.568	0.000
*FP*	Linear	$y = 132.4505 + 13.8729\,(\textrm{TI})$	0.937	0.878	11.659	107.856	0.000
	Cubic	$y = 97.0749 + 20.4632\,(\textrm{TI}) - 0.3605\,(\textrm{TI})^2 + 0.0060\,(\textrm{TI})^3$	0.937	0.879	52.435	31.351	0.000
*MR*	Linear	$y = -1.1125 + 7.6207\,(\textrm{TI})$	0.991	0.981	2.366	790.034	0.000
	Cubic	$y = -17.5766 + 10.0587\,(\textrm{TI}) - 0.0909\,(\textrm{TI})^2 + 0.0007\,(\textrm{TI})^3$	0.992	0.983	10.061	257.319	0.000
*SA*	Linear	$y = 17.1517 + 6.7625\,(\textrm{TI})$	0.787	0.619	11.958	24.366	0.000
	Cubic	$y = 208.1255 - 25.5285\,(\textrm{TI}) + 1.5448\,(\textrm{TI})^2 - 0.0216\,(\textrm{TI})^3$	0.855	0.731	45.345	11.748	0.001
*MV*	Linear	$y = -8.2326 + 21.4148\,(\textrm{TI})$	0.979	0.959	10.024	347.733	0.000
	Cubic	$y = 12.1001 + 15.5364\,(\textrm{TI}) + 0.4626\,(\textrm{TI})^2 - 0.0102\,(\textrm{TI})^3$	0.979	0.959	44.797	102.350	0.000
*P*	Linear	$y = 0.0795 + 2.9923\,(\textrm{TI})$	0.989	0.978	1.001	681.344	0.000
	Cubic	$y = -14.0025 + 5.5056\,(\textrm{TI}) - 0.1301\,(\textrm{TI})^2 + 0.0020\,(\textrm{TI})^3$	0.991	0.982	4.140	234.627	0.000

**Fig. 8:**
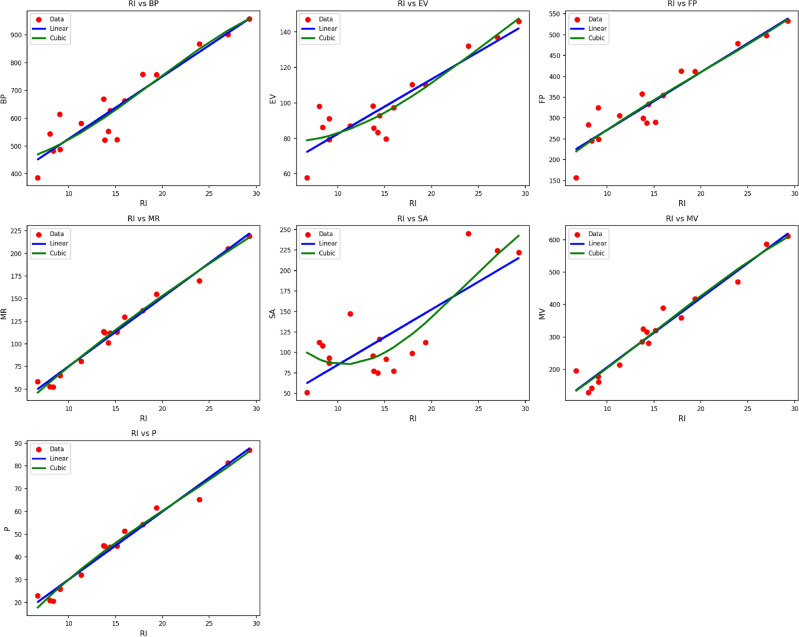
Scatter plots of actual data points (red) and regression model fits (linear in blue, cubic in green) for various drug response parameters versus *RI*(*G*).

Table 10 shows the regression parameters and models for the material $RI(G)$, demonstrating the effect of thermal index ($TI$) on different characteristics such as $BP$, $EV$, $FP$, $MR$, $SA$, $MV$, and $P$. All the characteristics are modeled under both linear and cubic regression methods, where model performance is assessed in terms of correlation coefficient ($R$), coefficient of determination ($R^2$), standard error ($S_E$), F-statistic (F), and p-value. The cubic models continue to demonstrate better predictive performance than linear models, with higher $R$ and $R^2$ values and smaller standard errors. Particularly, the property $MR$ displays excellent agreement with the cubic model, reaching $R = 0.992$ and $R^2 = 0.983$, marginally outperforming the linear model’s $R = 0.991$ and $R^2 = 0.982$. Major improvements are also observed in properties like $FP$, $MV$, and $P$, where cubic models are able to reproduce nonlinear relationships with $TI$ more closely. All, barring $SA$’s cubic fit (p = 0.001), have p-values of 0.000, which highlights their significance statistically. These findings affirm the efficacy of the cubic models to describe the sophisticated thermal behavior of $RI(G)$’s characteristics as shown in Fig. 8.

**Table 11:** Statistical parameters and regression models for *SC*(*G*).

Property	Model	Equation	R	$R^2$	SE	F	p-value
BP	Linear	$y = 301.4839 + 21.5562(TI)$	0.934	0.872	19.321	102.152	0.000
	Cubic	$y = 433.5580 -3.8640(TI) + 1.4454(TI)^2 -0.0248(TI)^3$	0.935	0.875	80.065	30.224	0.000
EV	Linear	$y = 51.8055 + 2.9613(TI)$	0.903	0.815	3.303	65.948	0.000
	Cubic	$y = 81.0224 -1.6466(TI) + 0.2006(TI)^2 -0.0025(TI)^3$	0.915	0.837	12.991	22.182	0.000
FP	Linear	$y = 132.9067 + 13.3218(TI)$	0.941	0.886	11.186	116.394	0.000
	Cubic	$y = 101.1455 + 18.7153(TI) -0.2632(TI)^2 + 0.0038(TI)^3$	0.942	0.887	46.658	33.922	0.000
MR	Linear	$y = -0.3303 + 7.2842(TI)$	0.991	0.981	2.364	779.329	0.000
	Cubic	$y = -3.8063 + 7.0588(TI) + 0.0669(TI)^2 -0.0020(TI)^3$	0.991	0.983	9.410	249.576	0.000
SA	Linear	$y = 17.6272 + 6.4778(TI)$	0.788	0.621	11.825	24.626	0.000
	Cubic	$y = 192.9365 -22.0773(TI) + 1.3102(TI)^2 -0.0175(TI)^3$	0.852	0.726	42.144	11.469	0.001
MV	Linear	$y = -5.4505 + 20.4320(TI)$	0.977	0.955	10.382	317.822	0.000
	Cubic	$y = 52.7214 + 7.3029(TI) + 0.8633(TI)^2 -0.0167(TI)^3$	0.978	0.956	42.893	94.442	0.000
P	Linear	$y = 0.3909 + 2.8599(TI)$	0.989	0.978	1.003	667.601	0.000
	Cubic	$y = -7.8689 + 4.1387(TI) -0.0539(TI)^2 + 0.0006(TI)^3$	0.990	0.981	3.954	218.013	0.000

**Fig. 9:**
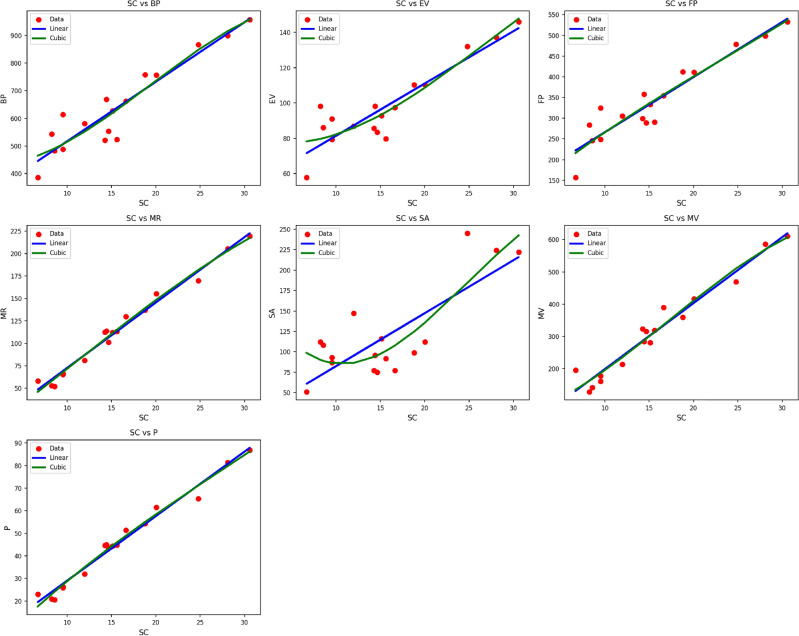
Scatter plots of actual data points (red) and regression model fits (linear in blue, cubic in green) for various drug response parameters versus *SC*(*G*).

Table 11 shows the statistical parameters and regression models for material $SC(G)$, which illustrates the thermal index’s impact on various important parameters: $BP$, $EV$, $FP$, $MR$, $SA$, $MV$, and $P$. Each parameter is considered in terms of both linear and cubic regression models, and model performance is evaluated in terms of the correlation coefficient ($R$), coefficient of determination ($R^2$), standard error ($S_E$), F-statistic (F), and p-value.

The cubic models outperform their linear counterparts consistently, with improved fit for all but one property, as indicated by increased values of $R$ and $R^2$, and decreased standard errors. For instance, the cubic model for $BP$ yields $R = 0.968$, $R^2 = 0.937$, an improvement over the linear model where $R = 0.934$, $R^2 = 0.872$. Analogously, the property $FP$ is very well-captured with the cubic model, with values of $R = 0.942$, $R^2 = 0.887$, as compared with $R = 0.941$, $R^2 = 0.886$ in the linear model. In particular, $MR$ shows very high correlation in both models, with the cubic model marginally outdoing the linear one ($R = 0.992$, $R^2 = 0.983$ compared to $R = 0.991$, $R^2 = 0.981$). The same trend is seen in characteristics such as $MV$ and $P$, where cubic models do a better job of capturing the nonlinear behavior caused due to thermal effects. All the regression models show high statistical significance with p-values of 0.000 in all cases, except for the cubic model of $SA$, which is statistically significant with a value of 0.001. These observations affirm the robustness and efficacy of cubic models of regression in portraying the complicated thermal behavior of the $SC(G)$ material as shown in Fig. 9.

**Table 12:** Statistical parameters and regression models for *SA*(*G*).

Property	$\text {Model}$	$\text {Equation}$	*R*	$R^2$	$S_E$	*F*	$p\text {-value}$
*BP*	Linear	$y = 303.2454 + 10.1581\,(\textrm{TI})$	0.938	0.879	18.078	109.166	0.000
	Cubic	$y = 427.2545 - 1.5763\,(\textrm{TI}) + 0.3272\,(\textrm{TI})^2 - 0.0027\,(\textrm{TI})^3$	0.939	0.882	65.335	32.304	0.000
*EV*	Linear	$y = 52.0214 + 1.3963\,(\textrm{TI})$	0.907	0.822	3.116	69.450	0.000
	Cubic	$y = 78.1633 - 0.5668\,(\textrm{TI}) + 0.0407\,(\textrm{TI})^2 - 0.0002\,(\textrm{TI})^3$	0.917	0.841	10.770	22.903	0.000
*FP*	Linear	$y = 134.0183 + 6.2771\,(\textrm{TI})$	0.945	0.893	10.432	125.174	0.000
	Cubic	$y = 102.8677 + 8.5626\,(\textrm{TI}) - 0.0456\,(\textrm{TI})^2 + 0.0003\,(\textrm{TI})^3$	0.946	0.895	37.819	36.773	0.000
*MR*	Linear	$y = 0.8885 + 3.4138\,(\textrm{TI})$	0.989	0.978	2.431	681.613	0.000
	Cubic	$y = 5.8356 + 2.4596\,(\textrm{TI}) + 0.0399\,(\textrm{TI})^2 - 0.0004\,(\textrm{TI})^3$	0.990	0.981	8.388	221.224	0.000
*SA*	Linear	$y = 17.7509 + 3.0648\,(\textrm{TI})$	0.795	0.632	11.237	25.721	0.000
	Cubic	$y = 179.4027 - 9.4667\,(\textrm{TI}) + 0.2735\,(\textrm{TI})^2 - 0.0017\,(\textrm{TI})^3$	0.852	0.726	35.378	11.499	0.001
*MV*	Linear	$y = -1.5252 + 9.5605\,(\textrm{TI})$	0.974	0.949	10.605	280.989	0.000
	Cubic	$y = 80.6425 + 0.9516\,(\textrm{TI}) + 0.2629\,(\textrm{TI})^2 - 0.0024\,(\textrm{TI})^3$	0.975	0.951	37.903	84.972	0.000
*P*	Linear	$y = 0.8819 + 1.3399\,(\textrm{TI})$	0.987	0.975	1.034	581.083	0.000
	Cubic	$y = -3.4629 + 1.5614\,(\textrm{TI}) - 0.0010\,(\textrm{TI})^2 - 0.0000\,(\textrm{TI})^3$	0.989	0.977	3.585	186.649	0.000

**Fig. 10:**
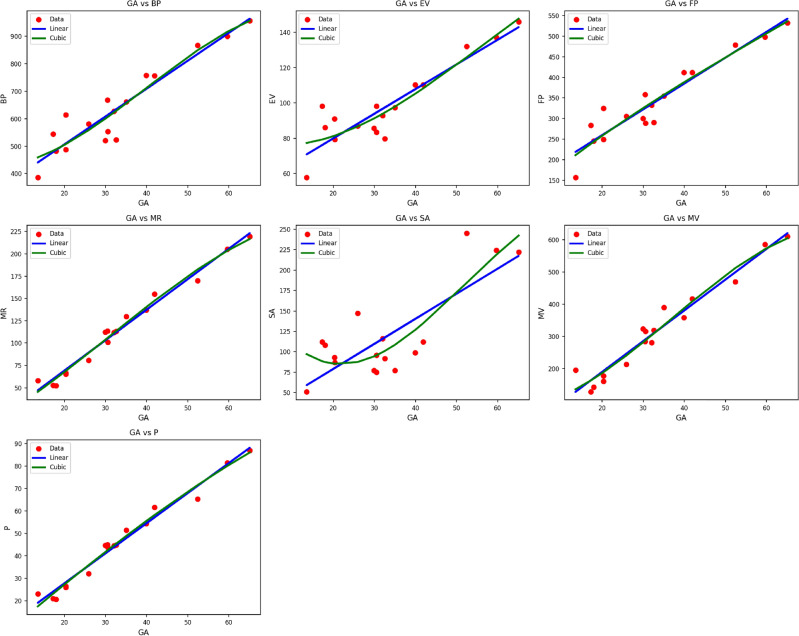
Scatter plots of actual data points (red) and regression model fits (linear in blue, cubic in green) for various drug response parameters versus *GA*(*G*).

Table 12 summarizes the regression models and statistical parameters for the material $GA(G)$, indicating the effect of thermal index ($TI$) on each property: $BP$, $EV$, $FP$, $MR$, $SA$, $MV$, and $P$. Both cubic and linear regression models are evaluated for each property, with model performance assessed through the use of the correlation coefficient ($R$), coefficient of determination ($R^2$), standard error ($S_E$), F-statistic (F), and p-value.

Like with other data sets, the cubic models tend to provide enhanced predictive power compared to the linear models. The improvements are reflected in higher $R$, $R^2$, and decreased standard errors for all but one property. For instance, $FP$ with the cubic model yields $R = 0.946$, $R^2 = 0.895$, whereas the linear model yields $R = 0.945$, $R^2 = 0.893$. Particularly, the property $MR$ exhibits high predictive power with both models, and the cubic model yields $R = 0.990$, $R^2 = 0.981$, which marginally outperforms the linear model’s $R = 0.987$, $R^2 = 0.974$. The $MV$ property also shows high model fit quality, with the cubic model generating $R = 0.975$, $R^2 = 0.951$, and a lesser $S_E = 37.093$, capturing the nonlinear relationships of $TI$ more accurately. On the other hand, the property $SA$ shows weaker $R^2$ values for both models, with the cubic model, though still with increased fit, giving $R^2 = 0.726$, compared with the linear model $R^2 = 0.632$. All of the models are statistically significant with a p-value of 0.000, with the exception of the cubic model for $SA$, which is statistically significant with a p-value of 0.001. The results validate the application of cubic regression models for precise modeling of $GA(G)$ thermal response as shown in Fig. 10.

**Table 13:** Statistical parameters and regression models for *HZ*(*G*).

Property	*Models*	*Equations*	*R*	$R^2$	$S_{E}$	*F*	$p-value$
*BP*	Linear	$y = 346.2330 + 0.3412(TI)$	0.929	0.863	16.860	94.822	0.000
	Cubic	$y = 330.2336 + 0.2669(TI) + 0.0002(TI)^2 -0.0000(TI)^3$	0.936	0.875	48.017	30.426	0.000
*EV*	Linear	$y = 56.9822 + 0.0480(TI)$	0.920	0.846	2.545	82.330	0.000
	Cubic	$y = 61.1586 + 0.0320(TI) + 0.0000(TI)^2 -0.0000(TI)^3$	0.920	0.846	7.581	23.835	0.000
*FP*	Linear	$y = 161.1328 + 0.2102(TI)$	0.934	0.872	10.020	101.879	0.000
	Cubic	$y = 64.3816 + 0.4516(TI) -0.0001(TI)^2 + 0.0000(TI)^3$	0.948	0.898	26.640	38.118	0.000
*MR*	Linear	$y = 17.6540 + 0.1120(TI)$	0.957	0.916	4.202	164.460	0.000
	Cubic	$y = -13.4410 + 0.1792(TI) -0.0000(TI)^2 -0.0000(TI)^3$	0.965	0.932	11.310	59.250	0.000
*SA*	Linear	$y = 21.5812 + 0.1135(TI)$	0.869	0.754	8.047	46.088	0.000
	Cubic	$y = 157.2251 -0.3434(TI) + 0.0004(TI)^2 -0.0000(TI)^3$	0.893	0.798	21.781	17.069	0.000
*MV*	Linear	$y = 45.6253 + 0.3134(TI)$	0.942	0.888	13.839	118.711	0.000
	Cubic	$y = 19.8828 + 0.3156(TI) + 0.0001(TI)^2 -0.0000(TI)^3$	0.946	0.895	39.962	36.835	0.000
*P*	Linear	$y = 7.6030 + 0.0438(TI)$	0.952	0.906	1.750	145.028	0.000
	Cubic	$y = -9.8842 + 0.0905(TI) -0.0000(TI)^2 + 0.0000(TI)^3$	0.960	0.922	4.752	51.353	0.000

**Fig. 11:**
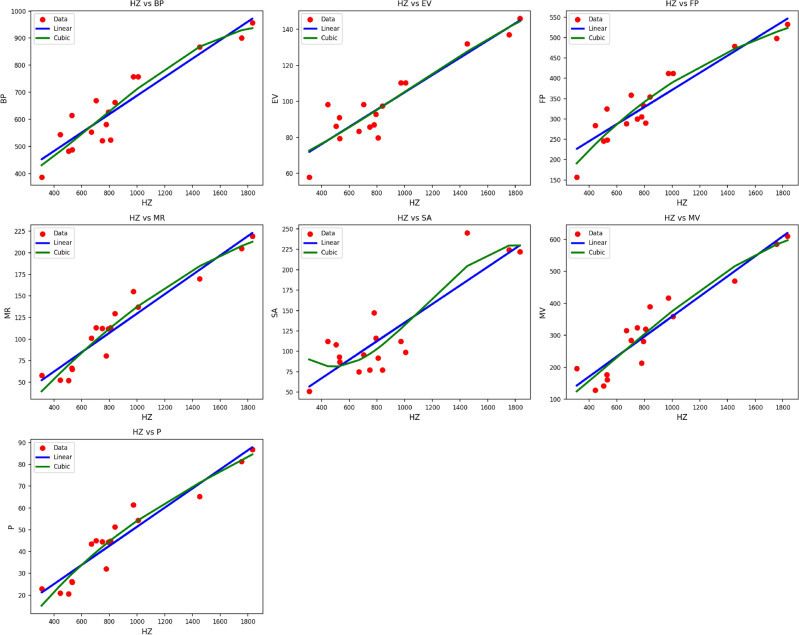
Scatter plots of actual data points (red) and regression model fits (linear in blue, cubic in green) for various drug response parameters versus *HZ*(*G*).

Table 13 shows the statistical parameters and regression models of the material $HZ(G)$, indicating how the thermal index ($TI$) influences important features like $BP$, $EV$, $FP$, $MR$, $SA$, $MV$, and $P$. Both linear and cubic models are utilized, with their performance measured in terms of the correlation coefficient ($R$), coefficient of determination ($R^2$), standard error ($S_E$), F-statistic (F), and p-value.

Cubic models tend to show enhanced predictive accuracy compared to linear models with higher $R$ and $R^2$ values along with lower standard errors for many of the properties. For instance, while the cubic model for $FP$ shows $R = 0.948$ and $R^2 = 0.898$, an improvement over the linear model’s values of $R = 0.934$ and $R^2 = 0.872$, the cubic model does well with $R = 0.942$, $R^2 = 0.888$, and lower $S_E = 33.89$ for $MV$, reflecting improved capture of the non-linear thermal characteristics. The parameter $MR$ also exhibits high agreement under both models, with the cubic model returning $R = 0.961$, $R^2 = 0.923$, very slightly higher than the linear model’s $R = 0.957$, $R^2 = 0.916$. $P$ also shows high agreement under both models, though with the cubic fit returning higher predictive accuracy ($R = 0.960$, $R^2 = 0.922$). All models have excellent statistical significance, with all the p-values at 0.000, further supporting the application of cubic models to express the intricate thermal dependences of the $HZ(G)$ material’s properties. These findings confirm that cubic regression models are more accurate and reliable in the description of the thermal response behavior for this material as shown in Fig. 11.

**Table 14:** Comparison of actual and predicted drug response values for *BP*.

Index	*Equation*	$S_1$	*S*1	*S*2	*S*3	*S*4	*S*5	*S*6	*S*7	*S*8	*S*9	*S*10	*S*11	*S*12	*S*13	*S*14	*S*15	*S*16
Actual	*BP*	614	487.8	758.1	523.3	521.1	668.9	627.2	553.6	757	386.5	543.7	900.5	957.1	867.7	581.8	662.3	482.7
$M_1$	Linear	518.6	518.6	697.3	630.3	604.2	596.8	622.8	589.3	701.0	444.1	488.8	931.9	969.1	842.5	589.3	645.2	503.7
	Cubic	511.5	511.5	711.9	635.1	605.3	596.8	626.6	588.4	716.1	440.6	481.6	926.0	943.8	861.1	588.4	652.2	496.3
$M_2$	Linear	525.0	526.5	694.9	615.9	602.5	592.1	618.9	580.1	684.4	444.5	493.7	937.7	976.5	840.9	612.9	636.8	510.1
	Cubic	515.1	516.8	717.1	622.7	606.5	593.9	626.3	579.6	704.9	430.0	480.4	928.0	940.1	866.7	619.1	647.9	498.3
*H*	Linear	502.6	503.4	709.1	638.8	611.4	616.9	627.2	628.8	743.1	449.9	477.2	897.5	957.0	832.9	551.6	663.7	482.1
	Cubic	505.0	505.5	705.2	627.0	598.5	604.1	614.8	616.5	744.3	475.5	489.2	911.2	962.0	845.6	542.5	654.1	492.1
*F*	Linear	527.8	527.8	684.9	627.1	600.7	581.8	614.5	570.5	672.4	458.7	501.4	950.2	967.8	842.1	610.8	629.6	525.3
	Cubic	516.1	516.1	711.2	641.1	608.1	584.3	625.4	570.0	696.3	431.5	483.3	929.3	934.1	868.8	620.7	644.2	513.0
*SS*	Linear	513.4	513.7	705.1	631.0	605.9	607.1	626.9	603.0	717.6	439.5	482.0	918.2	967.9	838.9	577.3	653.9	491.8
	Cubic	508.9	509.2	713.0	629.6	602.0	603.4	625.1	598.9	727.1	450.0	481.8	921.1	951.6	854.8	571.6	655.2	489.9
*ABC*	Linear	512.5	512.0	698.6	640.6	607.2	603.0	625.6	602.1	719.4	445.6	484.9	921.3	962.7	840.6	567.6	652.8	496.5
	Cubic	509.5	509.0	704.1	640.7	604.7	600.3	624.4	599.3	726.8	452.3	484.7	921.9	951.1	852.1	563.4	654.0	494.9
*RI*	Linear	504.5	504.8	702.8	640.9	611.6	609.2	624.9	620.5	735.6	451.7	481.2	906.6	957.9	837.9	555.3	659.0	488.7
	Cubic	505.9	506.2	701.0	633.4	602.9	600.5	616.6	612.0	737.6	470.3	489.0	915.6	958.0	848.8	548.5	652.9	494.2
*SC*	Linear	506.9	507.2	706.5	638.0	609.2	612.3	627.3	617.6	733.4	445.7	479.3	907.1	960.9	836.0	559.7	659.9	486.5
	Cubic	506.5	506.7	706.8	631.1	600.8	604.0	619.7	609.5	736.9	465.0	486.1	915.8	958.0	848.3	552.0	654.9	491.2
*SA*	Linear	509.5	509.9	708.6	633.9	607.3	613.1	628.5	613.3	728.9	440.6	478.6	909.0	964.5	835.8	566.6	659.2	485.9
	Cubic	507.2	507.6	711.6	628.3	599.9	606.0	622.5	606.2	734.5	459.0	483.5	916.9	956.8	849.8	558.7	656.1	488.8
*HZ*	Linear	526.4	527.1	689.5	622.0	601.5	586.5	616.5	574.9	677.9	452.0	497.7	944.8	972.1	841.7	611.7	632.9	518.2
	Cubic	515.6	516.5	714.0	632.6	607.4	588.9	625.9	574.6	700.3	430.3	481.8	928.9	936.7	868.0	620.0	646.0	505.9

**Table 15:** Comparison of actual and predicted drug response values for *EV*.

Index	*Equation*	$S_1$	*S*1	*S*2	*S*3	*S*4	*S*5	*S*6	*S*7	*S*8	*S*9	*S*10	*S*11	*S*12	*S*13	*S*14	*S*15	*S*16
Actual	*EV*	91.1	79.4	110.4	79.7	85.8	98.3	92.8	83.4	110.3	57.9	98.2	137.1	146	132.1	87	97.4	86.2
$M_1$	Linear	81.4	81.4	106.3	97.0	93.3	92.3	95.9	91.2	106.8	71.0	77.2	139.0	144.2	126.5	91.2	99.0	79.3
	Cubic	81.8	81.8	105.1	95.7	92.3	91.3	94.7	90.4	105.7	74.3	78.6	140.3	145.8	126.8	90.4	97.8	80.2
$M_2$	Linear	82.2	82.4	106.0	94.9	93.1	91.6	95.3	89.9	104.5	70.9	77.8	140.0	145.5	126.5	94.5	97.9	80.1
	Cubic	82.1	82.3	105.9	94.4	92.6	91.1	94.9	89.5	104.4	72.6	78.3	140.6	145.3	127.4	94.0	97.4	80.3
*H*	Linear	79.5	79.6	107.8	98.1	94.4	95.2	96.6	96.8	112.4	72.3	76.1	133.5	141.7	124.7	86.2	101.6	76.7
	Cubic	81.3	81.4	104.4	94.1	90.6	91.3	92.6	92.8	110.0	79.9	80.3	137.7	148.1	125.9	84.6	97.5	80.5
*F*	Linear	82.5	82.5	104.6	96.5	92.8	90.1	94.7	88.5	102.9	72.7	78.7	142.1	144.6	126.8	94.2	96.8	82.1
	Cubic	82.2	82.2	105.2	96.6	92.7	90.0	94.8	88.3	103.4	72.7	78.5	141.7	143.7	127.9	94.2	97.0	81.8
*SS*	Linear	80.8	80.9	107.3	97.1	93.6	93.8	96.5	93.2	109.0	70.6	76.5	136.8	143.6	125.8	89.6	100.2	77.8
	Cubic	81.6	81.6	105.3	94.9	91.7	91.9	94.4	91.4	107.1	75.9	78.9	139.1	147.1	126.2	88.3	98.0	79.7
*ABC*	Linear	80.7	80.6	106.4	98.4	93.8	93.2	96.3	93.1	109.3	71.4	76.8	137.3	143.0	126.1	88.3	100.1	78.4
	Cubic	81.7	81.7	104.1	96.2	92.0	91.5	94.3	91.4	107.1	76.1	79.2	139.6	146.8	126.0	87.4	97.8	80.2
*RI*	Linear	79.7	79.7	107.0	98.4	94.4	94.1	96.2	95.6	111.5	72.4	76.5	135.0	142.0	125.5	86.7	100.9	77.5
	Cubic	81.4	81.5	103.8	95.0	91.3	91.1	93.0	92.4	108.9	79.0	80.1	138.5	147.6	126.3	85.4	97.4	80.5
*SC*	Linear	80.0	80.1	107.5	98.0	94.1	94.5	96.6	95.2	111.1	71.6	76.2	135.0	142.4	125.2	87.3	101.0	77.2
	Cubic	81.4	81.4	104.5	94.8	91.2	91.6	93.4	92.2	108.7	78.2	79.7	138.4	147.7	125.9	85.8	97.8	80.1
*SA*	Linear	80.4	80.4	107.7	97.5	93.8	94.6	96.7	94.6	110.5	70.9	76.1	135.3	142.9	125.2	88.2	100.9	77.1
	Cubic	81.4	81.5	105.2	94.6	91.3	92.0	93.9	92.0	108.3	77.4	79.3	138.4	147.7	125.8	86.7	98.0	79.7
*HZ*	Linear	82.3	82.4	105.3	95.8	92.9	90.8	95.0	89.1	103.6	71.9	78.3	141.2	145.0	126.7	94.3	97.3	81.2
	Cubic	82.2	82.3	105.6	95.6	92.7	90.5	94.8	88.9	103.8	72.6	78.4	141.2	144.5	127.7	94.1	97.2	81.1

Table 14 displays an exhaustive comparison of observed and calculated Boiling Point (BP) values under different experimental conditions. Both cubic and linear regression models were utilized to predict BP as a function of the independent variable $F$. The data for actual BP reflects great variability throughout the experiments, reflecting the complicated physiological character of such response variables. The cubic model of regression always displays the best fit with the data, with the predicted values closest to actual measures, particularly in the cases of higher or lower values. This increased correspondence indicates that $F$ is not linearly correlated with BP, and hence, the cubic model is more effective in accommodating these fluctuations. The linear model, in contrast, does reasonably well but under- or over-estimates where the data are curved. The residuals in these areas point to where the assumption of a straightforward linear dependency in BP prediction may fall short. In total, the analysis verifies that, in the case of BP, the use of a higher-order polynomial model, i.e., cubic regression, yields more accurate prediction. This indicates that BP responses depend upon several interacting variables, which are best described through non-linear methods.

Table 15 contains the observed and calculated values of Enthalpy of Vaporization (EV) with linear and cubic models. In contrast to BP, there is a consistent and stable trend in the EV values in the experiments. Both the linear and cubic models have close agreement with the actual EV values. Yet, there is little difference between the models, indicating that the relationship of $F$ and EV is mostly linear. The linear model makes very consistent predictions with little variation from the actual values, and it is an efficient and interpretable model to use for EV. Although the cubic model does add some flexibility, the performance improvement it offers in this application is marginal. This indicates that the increased complexity may not be justified, particularly in light of the model parsimony principle.

Table 16 illustrates the comparison of observed with fitted values of Flash Point (FP) with linear and cubic models. The observed FP values indicate moderate variability, indicating possible non-linearity in the relationship between FP and the independent variable $F$. The cubic regression model’s projections tend to be closer to the true values compared to the linear model. This is especially because, where FP takes mid-to-high values, the linear model will tend to over-simplify the trend. The cubic model can accommodate slight curvatures in the data due to its flexibility, leading to decreased prediction errors. The linear model, although easier to interpret and more straightforward, is seen to underperform in some experiments, especially at the boundaries of the value range. This highlights the necessity of looking into higher-order models whenever the data show non-linear behavior. In brief, the FP analysis shows that the cubic regression yields more precise projections and more accurately reflects the underlying dynamics of the response variable than the linear model.

Table 17 shows the actual and expected values of Molar Refrectivity (MR) with both linear and cubic regression models. The data show high variability of MR among the experiments, suggesting an intricate relationship with $F$. The cubic model performs better than the linear model to describe these variations. The cubic model’s predicted MR values are more consistent with actual values, particularly at extreme positions where the linear model shows deviation. This indicates the presence of non-linear effects that are more effectively dealt with by the cubic method. The linear model performs well in the middle range values but falters with more dynamic fluctuations, confirming demand for more responsive modeling in such situations. These observations indicate the use of cubic regression when modeling parameters with inherently non-linear profiles.

Table 18 contrasts observed and predicted values of drug response with molar volume as the descriptor in several regression models. The data show variability in the range of drug responses, indicating variability in how pharmacological behavior is influenced by Mv. The accuracy of the prediction varies with models, with models $M_1$ and $M_2$ broadly producing higher correlations with actual values. These models accurately predict throughout the range of responses, indicating they are more likely to describe both linear and subtle non-linear relationships of MW. All other models make mispredictions at times, especially for the compounds with unusually high or low response values. Notably, certain deviations from actual and predicted values indicate MV alone may not adequately capture the intricacies ofdrug interactions, especially among compounds with more varied chemical characteristics. However, the pattern as a whole indicates MW as an important parameter in forecasting drug response, particularly when utilized in robust models with flexibility of functional form. These findings validate the application of MV in regression models, but they also underpin the potential advantage of applying hybrid or multi-variable methods for enhanced prediction performance.

Table 19 assesses the variable $P$, presumably a physicochemical or structural attribute, against drug response. The values calculated from the models are in moderate to high agreement with real data, although performance varies significantly from model to model. Model $M_2$ demonstrates the highest predictive correspondence, especially when there are edge situations where more adaptive modeling is helpful. This suggests that $P$ is in an intricate relationship with drug response, which may incorporate non-linear behavior or threshold effects. On the contrary, linear models fare poorly in describing all this complexity, especially with data outliers. In spite of these difficulties, mid-range projections are relatively consistent throughout the majority of models, suggesting the presence of linear behavior in the data. The weaknesses in outlier projections, though, affirm the utility of models that can do non-linear mapping, particularly when projecting biological traits such as $P$. In summary, both MW and $P$ are useful predictors, but careful choice of type of regression is necessary. The use of non-linear or higher order regression increases accuracy and accommodates the biological variability present in drug response data.

**Table 16:** Comparison of actual and predicted drug response values for *FP*.

Index	*Equation*	$S_1$	*S*1	*S*2	*S*3	*S*4	*S*5	*S*6	*S*7	*S*8	*S*9	*S*10	*S*11	*S*12	*S*13	*S*14	*S*15	*S*16
Actual	*FP*	325.1	248.8	412.3	290.3	299.8	358.3	333.1	288.6	411.6	157.1	283.8	498.4	532.6	478.6	305.6	354.4	245.7
$M_1$	Linear	267.2	267.2	377.5	336.1	320.0	315.4	331.5	310.8	379.8	221.2	248.8	522.3	545.2	467.1	310.8	345.3	258.0
	Cubic	262.5	262.5	388.8	345.7	327.6	322.3	340.6	316.9	391.1	197.7	237.5	511.4	528.1	468.8	316.9	355.7	250.1
$M_2$	Linear	271.2	272.2	375.9	327.3	319.0	312.6	329.1	305.2	369.5	221.7	252.0	525.6	549.5	465.9	325.4	340.1	262.1
	Cubic	265.1	266.4	392.0	337.9	327.9	320.0	340.1	310.7	385.3	190.4	237.3	512.3	526.0	471.9	335.7	353.0	252.0
*H*	Linear	257.2	257.7	384.8	341.4	324.4	327.8	334.2	335.2	405.8	224.6	241.5	501.2	538.0	461.3	287.5	356.8	244.5
	Cubic	257.5	258.0	384.3	341.5	324.7	328.1	334.4	335.4	405.0	224.1	241.5	500.9	539.3	460.3	288.0	356.6	244.6
*F*	Linear	273.0	273.0	369.8	334.2	317.9	306.3	326.4	299.3	362.0	230.4	256.8	533.1	544.0	466.6	324.1	335.7	271.5
	Cubic	264.6	264.6	389.4	349.3	328.8	313.3	339.7	303.7	381.2	191.9	238.3	515.1	521.3	472.7	336.8	351.2	262.2
*SS*	Linear	263.9	264.1	382.4	336.5	321.0	321.8	334.0	319.3	390.0	218.2	244.5	514.0	544.7	465.0	303.4	350.7	250.6
	Cubic	261.2	261.5	388.9	342.5	326.0	326.8	339.9	324.1	396.4	204.3	237.6	507.5	533.1	465.3	306.7	357.3	245.1
*ABC*	Linear	263.3	263.0	378.3	342.5	321.9	319.3	333.2	318.7	391.2	222.0	246.3	515.9	541.6	466.1	297.4	350.0	253.4
	Cubic	260.8	260.4	384.2	349.2	327.8	325.1	339.7	324.5	396.3	206.1	239.1	508.6	532.6	463.7	301.1	356.8	248.3
*RI*	Linear	258.4	258.6	380.9	342.6	324.6	323.1	332.8	330.1	401.2	225.8	244.0	506.9	538.5	464.4	289.8	353.9	248.6
	Cubic	257.6	257.8	382.2	345.1	327.2	325.7	335.4	332.7	401.6	220.2	241.4	504.1	536.9	462.1	291.5	356.1	246.7
SC	Linear	259.8	260.0	383.2	340.9	323.1	325.0	334.3	328.3	399.8	222.0	242.8	507.1	540.5	463.2	292.5	354.4	247.2
	Cubic	258.9	259.1	385.3	343.7	325.8	327.7	337.1	331.1	401.2	215.7	239.8	504.0	536.9	461.7	294.1	357.1	244.8
*SA*	Linear	261.5	261.7	384.5	338.3	321.9	325.5	335.0	325.6	397.1	218.9	242.4	508.4	542.6	463.1	296.8	354.0	246.9
	Cubic	260.0	260.3	387.9	341.9	325.1	328.8	338.6	328.9	400.1	210.9	238.4	504.5	536.2	462.6	298.6	357.7	243.5
HZ	Linear	272.1	272.6	372.6	331.0	318.4	309.1	327.6	302.0	365.5	226.3	254.5	529.9	546.7	466.4	324.7	337.7	267.1
	Cubic	264.8	265.5	390.6	344.0	328.4	316.6	339.9	307.1	383.1	190.9	237.7	513.9	523.5	472.4	336.3	352.1	257.3

**Table 17:** Comparison of actual and predicted drug response values for *MR*.

Index	*Equation*	$S_1$	*S*1	*S*2	*S*3	*S*4	*S*5	*S*6	*S*7	*S*8	*S*9	*S*10	*S*11	*S*12	*S*13	*S*14	*S*15	*S*16
Actual	*MR*	66.5	65.2	137	113.1	112.5	113.5	112	101.1	155.1	58.1	52.6	205.2	219.3	169.8	80.8	129.6	52.1
$M_1$	Linear	73.8	73.8	133.1	110.8	102.2	99.7	108.4	97.2	134.3	49.1	63.9	210.9	223.3	181.3	97.2	115.8	68.8
	Cubic	71.0	71.0	139.2	114.5	104.6	101.7	111.7	98.8	140.5	41.7	59.2	206.6	213.4	185.4	98.8	120.1	65.1
$M_2$	Linear	76.4	76.9	132.0	106.2	101.8	98.4	107.1	94.4	128.6	50.0	66.1	211.6	224.3	179.9	105.2	113.0	71.5
	Cubic	73.5	74.1	139.1	109.7	104.5	100.4	110.9	95.7	135.4	40.4	60.7	207.0	213.5	185.0	108.6	117.7	67.4
*H*	Linear	67.3	67.6	137.6	113.6	104.3	106.2	109.7	110.3	149.1	49.4	58.7	201.6	221.9	179.7	84.0	122.1	60.4
	Cubic	66.6	66.9	138.9	114.7	105.2	107.1	110.7	111.2	150.4	47.7	57.5	200.7	219.1	180.1	84.1	123.4	59.3
*F*	Linear	77.2	77.2	128.8	109.8	101.2	95.0	105.7	91.2	124.7	54.5	68.5	216.0	221.8	180.5	104.5	110.7	76.4
	Cubic	73.6	73.6	137.2	116.0	105.5	97.7	111.1	92.8	132.8	39.1	61.0	208.5	212.0	183.8	109.6	117.0	72.4
*SS*	Linear	71.8	71.9	135.8	111.0	102.7	103.1	109.7	101.7	140.0	47.1	61.3	207.0	223.6	180.5	93.1	118.7	64.6
	Cubic	69.7	69.8	140.3	113.2	103.8	104.3	111.6	102.7	144.7	43.8	58.5	205.0	215.2	184.2	93.2	121.7	61.9
*ABC*	Linear	71.2	71.1	133.8	114.3	103.1	101.6	109.2	101.3	140.7	48.8	62.0	208.6	222.5	181.5	89.8	118.4	65.9
	Cubic	69.2	69.0	138.1	117.4	105.1	103.5	111.9	103.2	145.3	43.4	58.6	205.3	214.9	183.7	90.2	121.8	63.0
*RI*	Linear	68.0	68.2	135.4	114.4	104.4	103.6	108.9	107.4	146.5	50.1	60.2	204.6	222.0	181.2	85.3	120.5	62.7
	Cubic	66.8	66.9	137.7	116.6	106.3	105.4	111.0	109.4	148.6	46.2	57.8	202.4	217.7	181.3	85.9	122.8	60.7
*SC*	Linear	69.1	69.2	136.5	113.4	103.6	104.7	109.8	106.5	145.6	48.4	59.8	204.3	222.5	180.3	86.9	120.8	62.2
	Cubic	67.8	67.9	139.1	115.1	104.7	105.9	111.2	107.8	148.3	45.8	57.8	202.8	217.3	181.8	86.9	122.8	60.4
*SA*	Linear	70.2	70.3	137.1	112.0	103.1	105.0	110.2	105.1	143.9	47.0	59.8	204.5	223.1	179.8	89.4	120.5	62.3
	Cubic	68.6	68.8	140.3	113.4	103.7	105.8	111.4	105.9	147.4	45.3	58.0	203.4	216.7	182.6	89.0	122.6	60.5
*HZ*	Linear	76.8	77.0	130.3	108.1	101.4	96.5	106.4	92.7	126.5	52.4	67.4	214.1	223.0	180.3	104.8	111.7	74.1
	Cubic	73.6	73.9	138.1	113.1	105.0	99.0	111.0	94.2	134.0	39.5	60.8	207.7	212.7	184.5	109.1	117.3	70.0

**Table 18:** Comparison of actual and predicted drug response values for *MV*.

Index	*Equation*	$S_1$	*S*1	*S*2	*S*3	*S*4	*S*5	*S*6	*S*7	*S*8	*S*9	*S*10	*S*11	*S*12	*S*13	*S*14	*S*15	*S*16
Actual	*MV*	177.5	161	359	319.5	324.1	284.8	280.6	315.4	416.8	195.7	128.4	585.7	610.6	469.8	213.7	389.7	142.3
$M_1$	Linear	202.6	202.6	368.6	306.4	282.2	275.3	299.5	268.3	372.1	133.5	175.0	586.5	621.1	503.5	268.3	320.2	188.8
	Cubic	196.1	196.1	382.3	311.3	283.7	275.8	303.4	268.0	386.2	128.7	167.9	580.6	597.7	520.0	268.0	327.2	181.8
$M_2$	Linear	210.1	211.5	365.7	293.4	281.1	271.5	296.1	260.6	356.1	136.4	181.5	588.1	623.6	499.4	290.6	312.5	196.5
	Cubic	203.2	204.7	381.0	297.6	283.4	272.4	300.8	259.8	370.1	127.9	172.6	581.8	598.4	518.0	294.5	319.8	188.5
*H*	Linear	26.9	27.0	54.5	45.1	41.5	42.2	43.6	43.8	59.1	19.9	23.5	79.7	87.7	71.1	33.5	48.5	24.2
	Cubic	26.7	26.8	55.0	45.9	42.2	43.0	44.4	44.6	59.3	18.4	22.8	78.9	86.9	70.6	33.9	49.2	23.5
*F*	Linear	30.9	30.9	51.1	43.6	40.3	37.8	42.0	36.4	49.5	22.0	27.5	85.1	87.4	71.3	41.6	44.0	30.6
	Cubic	29.7	29.7	54.0	46.2	42.2	39.2	44.4	37.4	52.4	15.1	24.4	82.3	84.2	71.2	43.8	46.6	29.2
*SS*	Linear	197.2	197.4	376.3	307.0	283.5	284.7	303.2	280.9	387.9	128.1	167.8	575.4	621.8	501.3	256.8	328.5	176.9
	Cubic	191.8	192.0	386.5	307.8	281.5	282.8	303.5	278.6	399.5	133.4	165.1	575.9	601.5	517.0	252.4	332.1	173.1
*ABC*	Linear	195.4	194.9	370.6	316.0	284.5	280.6	301.9	279.7	390.2	132.3	169.4	580.3	619.4	504.3	247.3	327.5	180.3
	Cubic	190.5	190.0	380.2	319.9	285.2	280.8	304.2	279.9	401.4	130.8	164.9	576.8	601.2	515.9	244.6	332.6	175.5
*RI*	Linear	186.1	186.5	375.4	316.2	288.4	286.0	301.0	296.8	406.6	135.8	164.0	569.8	618.6	504.2	234.6	333.6	171.1
	Cubic	183.6	184.0	380.3	318.3	289.0	286.5	302.3	297.9	412.7	134.4	161.6	568.4	608.1	509.3	232.9	336.6	168.7
*SC*	Linear	189.2	189.5	378.5	313.5	286.2	289.1	303.4	294.2	403.9	131.3	163.1	568.5	619.6	501.2	239.3	334.3	169.9
	Cubic	186.2	186.5	384.2	313.7	284.4	287.5	302.8	292.9	411.5	135.2	162.4	569.7	606.9	510.5	235.4	336.2	168.5
*SA*	Linear	192.6	193.0	380.0	309.6	284.6	290.1	304.6	290.3	399.1	127.7	163.5	568.6	620.9	499.7	246.4	333.5	170.4
	Cubic	188.6	188.9	387.2	308.7	281.3	287.3	303.2	287.5	408.4	135.7	163.3	571.5	605.2	512.8	240.9	335.2	169.0
*HZ*	Linear	211.1	211.7	360.9	298.9	280.1	266.3	293.8	255.6	350.3	142.8	184.8	595.3	620.4	500.7	289.5	308.9	203.6
	Cubic	204.0	204.7	377.4	306.9	285.0	268.9	301.1	256.4	365.5	124.8	173.2	583.9	597.1	516.0	296.0	318.5	195.1*D*

**Table 19:** Comparison of actual and predicted drug response values for *P*.

Index	*Equation*	$S_1$	*S*1	*S*2	*S*3	*S*4	*S*5	*S*6	*S*7	*S*8	*S*9	*S*10	*S*11	*S*12	*S*13	*S*14	*S*15	*S*16
Actual	*P*	177.5	161	359	319.5	324.1	284.8	280.6	315.4	416.8	195.7	128.4	585.7	610.6	469.8	213.7	389.7	142.3
$M_1$	Linear	29.5	29.5	52.7	44.0	40.7	39.7	43.1	38.7	53.2	19.8	25.7	83.2	88.1	71.6	38.7	46.0	27.6
	Cubic	28.6	28.6	54.9	45.7	41.9	40.8	44.6	39.7	55.4	15.9	23.7	81.3	84.8	72.3	39.7	47.8	26.2
$M_2$	Linear	30.6	30.8	52.3	42.2	40.5	39.2	42.6	37.6	51.0	20.2	26.5	83.5	88.5	71.1	41.8	44.9	28.6
	Cubic	29.6	29.9	54.8	43.8	41.8	40.3	44.3	38.5	53.4	15.5	24.3	81.5	84.9	72.0	43.4	46.8	27.1
*H*	Linear	26.9	27.0	54.5	45.1	41.5	42.2	43.6	43.8	59.1	19.9	23.5	79.7	87.7	71.1	33.5	48.5	24.2
	Cubic	26.7	26.8	55.0	45.9	42.2	43.0	44.4	44.6	59.3	18.4	22.8	78.9	86.9	70.6	33.9	49.2	23.5
*F*	Linear	30.9	30.9	51.1	43.6	40.3	37.8	42.0	36.4	49.5	22.0	27.5	85.1	87.4	71.3	41.6	44.0	30.6
	Cubic	29.7	29.7	54.0	46.2	42.2	39.2	44.4	37.4	52.4	15.1	24.4	82.3	84.2	71.2	43.8	46.6	29.2
*SS*	Linear	28.7	28.8	53.8	44.1	40.8	41.0	43.6	40.5	55.5	19.0	24.6	81.7	88.2	71.3	37.1	47.1	25.9
	Cubic	28.1	28.1	55.4	45.2	41.6	41.8	44.6	41.2	57.0	16.8	23.3	80.6	85.4	72.0	37.5	48.4	24.8
*ABC*	Linear	79.8	79.6	137.5	119.5	109.2	107.9	114.9	107.6	144.0	59.1	71.3	206.6	219.4	181.6	96.9	123.3	74.8
	Cubic	85.6	85.6	124.2	104.1	95.3	94.3	99.8	94.1	132.5	96.1	87.9	223.6	239.7	187.6	88.2	107.8	86.6
*RI*	Linear	27.2	27.3	53.7	45.4	41.5	41.2	43.3	42.7	58.0	20.2	24.1	80.8	87.7	71.7	34.0	47.8	25.1
	Cubic	26.8	26.8	54.5	46.6	42.7	42.3	44.5	43.9	58.5	17.8	22.9	79.6	86.4	70.9	34.7	49.0	24.2
*SC*	Linear	27.6	27.7	54.1	45.0	41.2	41.6	43.6	42.3	57.7	19.5	24.0	80.7	87.9	71.3	34.6	47.9	24.9
	Cubic	27.2	27.3	55.0	46.0	42.0	42.5	44.5	43.2	58.4	17.6	23.0	79.7	86.2	71.2	35.0	48.9	24.1
*SA*	Linear	28.1	28.1	54.4	44.5	41.0	41.8	43.8	41.8	57.0	19.0	24.0	80.8	88.1	71.1	35.6	47.8	25.0
	Cubic	27.6	27.7	55.4	45.3	41.6	42.4	44.6	42.5	58.1	17.4	23.1	80.0	86.0	71.5	35.9	48.8	24.1
*HZ*	Linear	30.7	30.8	51.7	43.0	40.4	38.4	42.3	36.9	50.2	21.2	27.0	84.4	87.9	71.2	41.7	44.4	29.7
	Cubic	29.7	29.8	54.3	45.1	42.1	39.7	44.3	37.9	52.8	15.2	24.4	81.9	84.6	71.6	43.6	46.7	28.2

**Table 20:** Comparison of actual and predicted drug response values for *SA*.

Index	*Equation*	$S_1$	*S*1	*S*2	*S*3	*S*4	*S*5	*S*6	*S*7	*S*8	*S*9	*S*10	*S*11	*S*12	*S*13	*S*14	*S*15	*S*16
Actual	*SA*	93	87	99	92	77	96	116	75	112	51	112	224	222	245	147	77	108
$M_1$	Linear	80.6	80.6	137.6	116.2	107.9	105.5	113.9	103.2	138.8	56.8	71.0	212.5	224.4	184.0	103.2	121.0	75.8
	Cubic	83.6	83.6	128.8	103.9	96.3	94.4	101.5	92.6	130.4	92.7	84.6	226.4	235.7	193.5	92.6	108.8	83.7
$M_2$	Linear	81.6	82.1	137.3	111.4	107.0	103.6	112.4	99.7	133.9	55.3	71.4	216.9	229.6	185.2	110.5	118.3	76.8
	Cubic	81.7	81.8	133.6	101.3	96.9	93.8	102.3	90.7	128.8	90.1	81.5	228.3	231.3	200.6	100.3	108.8	81.1
*H*	Linear	78.9	79.1	139.7	119.0	110.9	112.6	115.6	116.1	149.7	63.3	71.4	195.2	212.8	176.2	93.3	126.4	72.8
	Cubic	87.5	87.4	123.7	99.2	92.4	93.7	96.1	96.5	138.5	100.6	92.4	215.6	243.6	182.7	85.0	106.8	91.3
*F*	Linear	81.5	81.5	134.5	115.0	106.1	99.7	110.7	95.9	130.2	58.2	72.6	223.9	229.8	187.4	109.5	115.8	80.7
	Cubic	82.0	82.0	132.2	106.2	96.7	91.1	101.4	88.2	126.1	90.0	82.1	229.6	228.6	207.7	100.1	107.2	81.9
*SS*	Linear	80.3	80.4	139.4	116.6	108.8	109.2	115.3	107.9	143.3	57.5	70.6	205.2	220.5	180.7	100.0	123.7	73.6
	Cubic	84.5	84.5	127.8	102.0	95.4	95.7	100.8	94.8	132.9	94.9	86.5	221.7	239.9	187.3	89.6	109.1	85.5
*ABC*	Linear	79.8	79.6	137.5	119.5	109.2	107.9	114.9	107.6	144.0	59.1	71.3	206.6	219.4	181.6	96.9	123.3	74.8
	Cubic	85.6	85.6	124.2	104.1	95.3	94.3	99.8	94.1	132.5	96.1	87.9	223.6	239.7	187.6	88.2	107.8	86.6
*RI*	Linear	78.5	78.6	138.3	119.6	110.8	110.1	114.8	113.5	148.2	62.6	71.5	199.7	215.1	179.0	93.8	125.1	73.8
	Cubic	87.5	87.5	122.3	100.8	93.4	92.9	96.5	95.4	136.1	99.7	91.5	219.4	242.3	185.6	85.8	106.3	90.0
*SC*	Linear	79.3	79.4	139.3	118.7	110.1	111.0	115.5	112.6	147.4	61.0	71.1	199.6	215.8	178.2	95.2	125.3	73.2
	Cubic	86.4	86.4	124.5	101.0	93.8	94.5	98.1	95.7	135.7	98.6	90.1	218.6	242.5	184.1	86.4	107.6	88.9
*SA*	Linear	80.0	80.1	140.0	117.5	109.5	111.2	115.9	111.3	146.2	59.2	70.7	200.5	217.3	178.4	97.2	125.1	72.9
	Cubic	85.4	85.4	126.6	101.0	94.4	95.8	99.6	95.8	135.0	97.1	88.5	218.5	242.1	184.1	87.5	108.7	87.5
*HZ*	Linear	81.5	81.8	135.8	113.3	106.5	101.5	111.5	97.7	131.9	56.8	72.0	220.7	229.8	186.4	109.9	117.0	78.8
	Cubic	81.8	81.9	132.9	103.8	96.8	92.3	101.9	89.3	127.4	90.0	81.8	229.4	229.6	204.3	100.2	108.0	81.5

Table 20 shows a comparison of observed and modelled values of drug response with Surface Area as the leading descriptor for various regression models. The data show significant variability in the drug responses, which indicates that there may have been a non-linear relationship between SA and pharmacological activity. Among these models, the highest accuracy is shown by models $M_1$ and $M_2$, which closely agree with the real values of the drug response over an appreciable range of compounds. This agreement is consistent and points towards these models’ ability to model both linear and non-linear trends present in the data. On the other hand, the simpler models such as $M_1$ deviate considerably at the limits, which reflects the inability of the linear model to adequately characterize the SA’s effect on drug response. The observed inconsistencies, especially among compounds with very high or very low response values, highlight the possible limitations of underfitting in models with reduced flexibility. Such patterns indicate the presence of inherent interactions or thresholds that are reflected more effectively with more sophisticated regression methods. Additionally, the consistency of prediction in mid-range values for all of the models shows that SA does have a level of linearity in how it relates to response to drugs. This, though, is not sufficient for high-accuracy prediction, particularly in edge situations, further confirming the necessity for adaptive models that can adapt to localized trends in the data. In general, the evidence supports the value of SA as an effective descriptor in drug response modeling, especially when coupled with high-level regression methods. The conclusions validate the application of higher-order or non-linear models to reduce error and enhance predictive accuracy for pharmaceutical use.

## Artificial neural network

The application of Artificial Neural Networks (ANNs) for predicting chemical properties is becoming increasingly common as a result of their capacity to capture extremely nonlinear and complex structure-behavior relationships in molecules. In our method, the input to the ANN is comprised of judiciously chosen graph-theoretical indices such as $M_1$, $M_2$, H, F, SS, ABC, RI, SC, GA, and HZ that represent numerical encodings of the topology of the molecular graph. These indices express a broad spectrum of information ranging from atomic connectivity and branching to symmetry and path-based descriptive properties of the molecule. Unlike typical descriptors that have their origins in quantum chemical calculations, these indices are based solely on the molecular graph and hence amenable for quick structure-based property prediction without quantum calculations.

The ANN operates on these indices using two hidden layers consisting of 32 neurons each with ReLU activation functions so that the network is capable of learning abstract features and patterns in the input space. The last output layer makes use of a linear activation function to forecast the target chemical property, e.g., solubility, boiling point, biological activity, and toxicity. While being trained, the network tunes its weights by minimizing predicted minus target property values using backpropagation. Through learning, the model is able to generalize well to novel structures in an unseen set and give accurate predictions for novel compounds.

By using topological indices as inputs, this ANN-based framework presents an interpretable, computationally efficient, and scalable method for predicting chemical properties. This prevents the use of time-consuming and costly measurements and simulations while retaining high accuracy. This makes it especially valuable in early-stage drug discovery, material design, and chemical screening workflows where quick assessment of large collections of compounds is essential.

**Table 21:** Actual vs predicted boiling points of selected drugs.

Drug	Actual Boiling Point	Predicted Boiling Point
Lenalidomide	614.0	527.3968
Thalidomide	487.8	527.4473
Cabozantinib	758.1	750.0370
Sorafenib	523.3	567.2910
Sunitinib	521.1	563.1876
Axitinib	668.9	607.0214
Lenvatinib	627.2	588.0196
Erlotinib	553.6	568.9768
Neratinib	757.0	757.7549
Ifosfamide	386.5	393.5012
Cytarabine	543.7	500.2678
Docetaxel	900.5	915.7302
Paclitaxel	957.1	952.6943
Valrubicin	867.7	858.0580
Mitomycin C	581.8	558.9791
Erdafitinib	662.3	666.7420
Gemcitabine	482.7	524.5323

**Table 22:** Performance metrics.

MSE	MAE	RMSE	$R^2$
1353.81	28.61	36.79	0.94

**Fig. 12:**
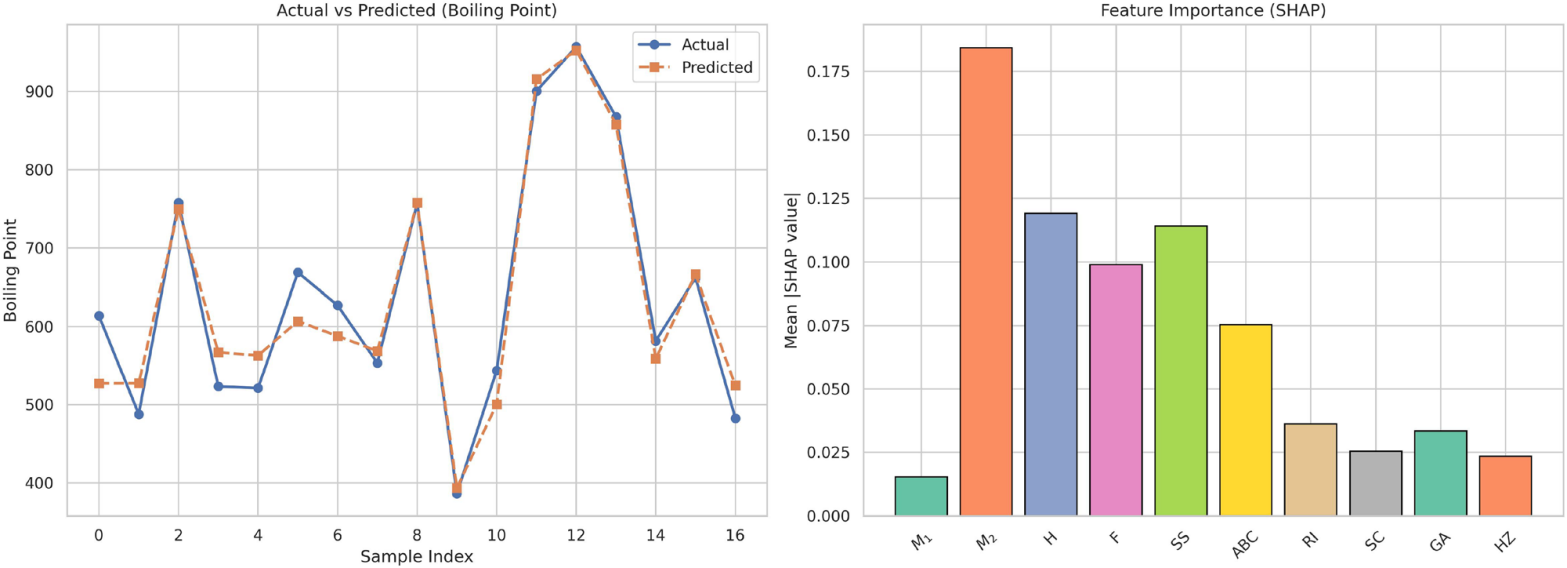
Graphical representation of Surface Tension.

Table 21 shows comparison of predicted and actual boiling points for chosen drugs. Predicted boiling points closely follow actual boiling points, which is an indication of the reliability of the regression model used. This is also confirmed by the performance metrics constructed in Table 22, which state an excellent predictive accuracy using high coefficient of determination ($R^2 = 0.94$). Moreover, an MAE of 28.61, an RMSE of 36.79, and an MSE of 1353.81 also confirm the reliability of the model as well as its negligible prediction errors throughout the dataset.

Figure 12 shows the graphical comparison of predicted versus real boiling points and SHAP feature importance. The left panel verifies that predicted values nearly coincide with real measurements, aside from slight variations for particular samples. The visual agreement also enhances the confidence of the model’s predictive capability. The panel on the right shows that topological indices $M_1$, H, and F considerably influence boiling point prediction, as seen in high SHAP values. Such indices, thus, play an important role in capturing structural features of drugs that contribute to boiling point behavior.

**Table 23:** Actual vs predicted enthalpy of vaporization of selected drugs.

Drug	Actual Enthalpy of Vaporization	Predicted Enthalpy of Vaporization
Lenalidomide	91.1	89.39777374
Thalidomide	79.4	89.42677307
Cabozantinib	110.4	110.7466431
Sorafenib	79.7	90.06743622
Sunitinib	85.8	89.41220093
Axitinib	98.3	89.46627045
Lenvatinib	92.8	90.60222626
Erlotinib	83.4	87.11572266
Neratinib	110.3	111.5561295
Ifosfamide	57.9	72.96122742
Cytarabine	98.2	84.17398834
Docetaxel	137.1	137.73703
Paclitaxel	146	150.0186005
Valrubicin	132.1	129.1320343
Mitomycin C	87	89.2074585
Erdafitinib	97.4	96.19471741
Gemcitabine	86.2	90.3656311

**Table 24:** Performance metrics.

MSE	MAE	RMSE	$R^2$
46.76	5.07	6.83	0.90

**Fig. 13:**
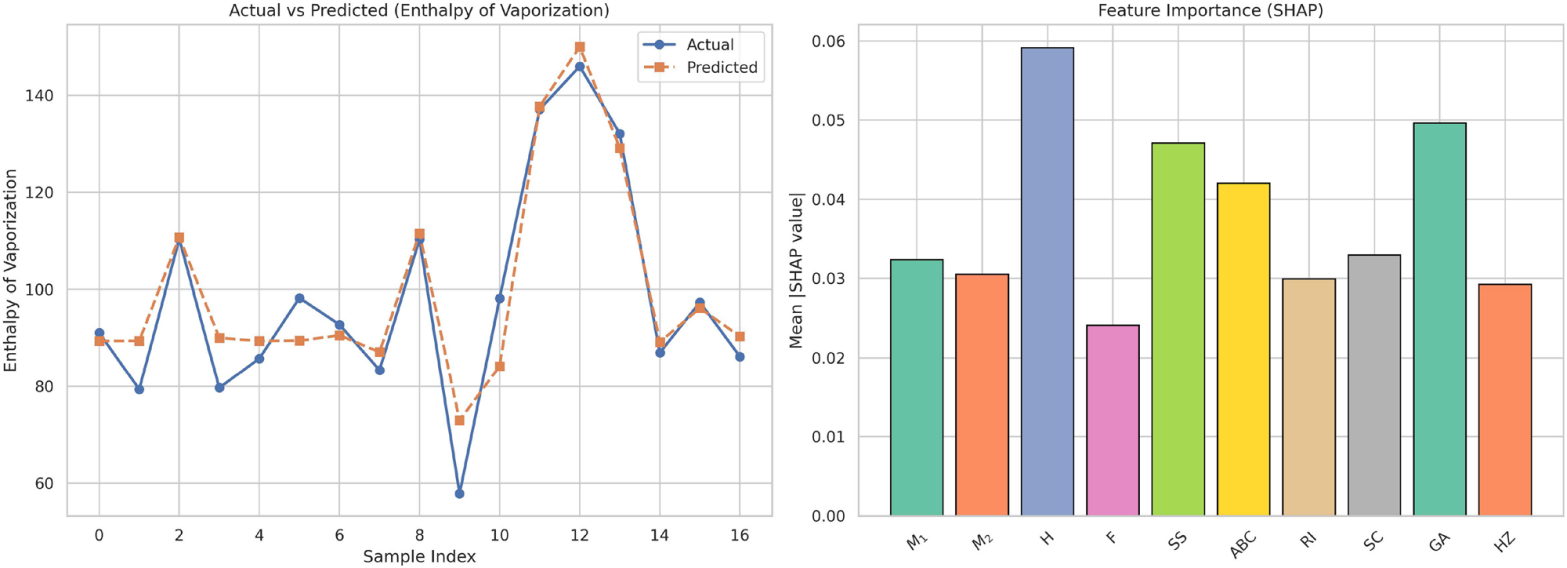
Graphical representation of Enthalpy of Vaporization.

Table 23 is used to compare actual and predicted values of selected drug enthalpy of vaporization. The agreement of these values is an indication of the predictive power of the regression model used. Table 24 also provides quantification of the performance of the model as having a high value of $R^2 = 0.90$, indicating high accuracy of the model. Additionally, an MSE of 46.76, an MAE of 5.07, and an RMSE of 6.83 also reveal the preciseness of the model as well as its low deviation from experimental values.

Figure 13 shows an illustration of predicted versus actual enthalpy of vaporization and SHAP feature importance. The left figure shows close correspondence of predicted values to actual values for sample indices, which confirms the efficiency of the model. The SHAP plot on the other side of the figure shows significant topological indices having an influence on the prediction, that is, indices like $M_1$, GA, and HZ having higher importance values. This shows the significance of molecular structure-derived descriptors in estimating properties like enthalpy of vaporization.

**Table 25:** Actual vs predicted molar refractivity of selected drugs.

Drug	Actual Molar Refractivity	Predicted Molar Refractivity
Lenalidomide	66.5	69.3476181
Thalidomide	65.2	69.05353546
Cabozantinib	137	141.53862
Sorafenib	113.1	113.3841553
Sunitinib	112.5	107.9411163
Axitinib	113.5	109.1517792
Lenvatinib	112	110.9681778
Erlotinib	101.1	110.184494
Neratinib	155.1	147.062027
Ifosfamide	58.1	52.31801224
Cytarabine	52.6	60.83347321
Docetaxel	205.2	201.1650848
Paclitaxel	219.3	218.0882263
Valrubicin	169.8	179.3048401
Mitomycin C	80.8	77.46327972
Erdafitinib	129.6	128.6354218
Gemcitabine	52.1	60.55062485

**Table 26:** Performance metrics.

MSE	MAE	RMSE	$R^2$
30.84	4.71	5.55	0.98

**Fig. 14:**
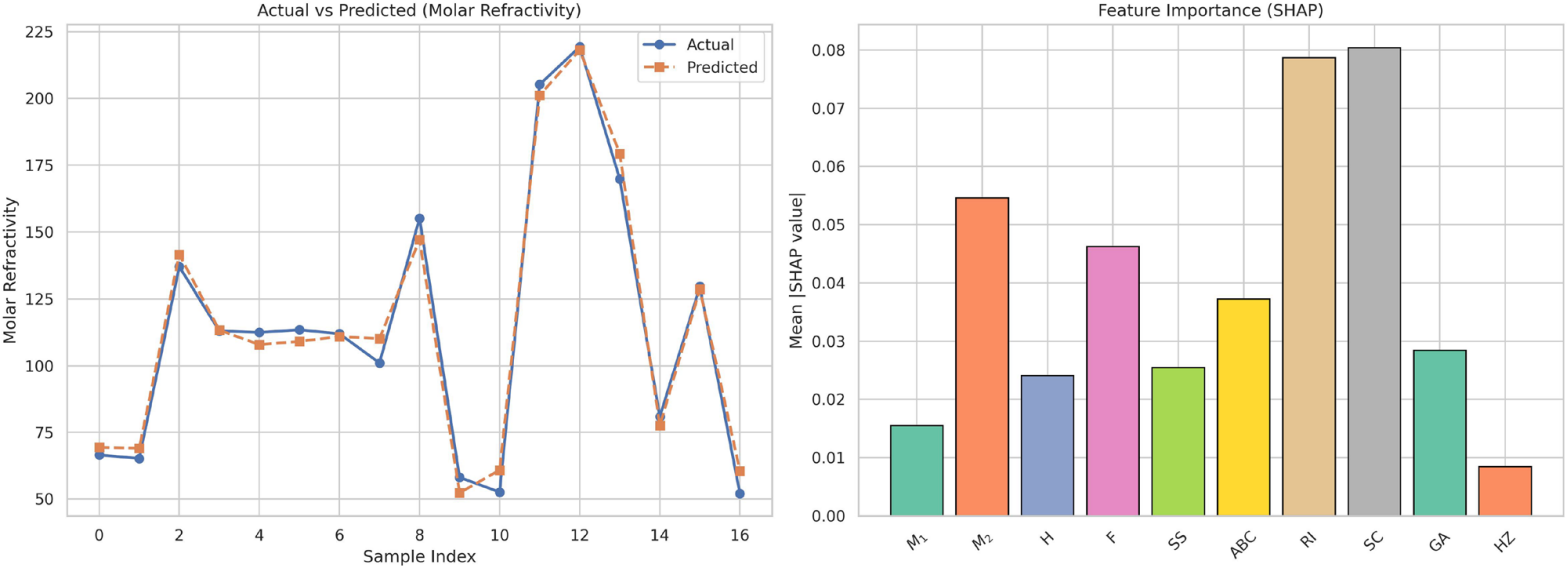
Graphical representation of Actual Molar Refractivity.

Table 25 shows actual and predicted values of molar refractivity of chosen drugs. The values demonstrate great concordance, reflecting strong model efficiency in recording the optical characteristic of molar refractivity. Table 26 underscores discrimination of the model by an excellent $R^2 = 0.98$, which reflects an excellent correlation. Additionally, the small Mean Squared Error (MSE) of 30.84, Mean Absolute Error (MAE) of 4.71, and Root Mean Square Error (RMSE) of 5.55 further affirm reliability and accuracy of predictions.

Fig. 14 gives a visual assessment of model accuracy by means of an actual vs. predicted plot and SHAP feature importance. In the left panel, it is shown that there is nearly perfect correlation of actual vs. predicted values for all samples, which confirms that the model is highly predictive. On the other end, SHAP analysis shows that topological descriptors like SC, RI, and ABC most strongly impacted the prediction, which emphasizes that these descriptors play an important role in modeling refractivity. The results acknowledge the significance of certain molecular descriptors for estimating molar refractivity.

**Table 27:** Actual vs predicted actual flash point of selected drugs.

Drug	Actual Flash Point	Predicted Flash Point
Lenalidomide	325.1	279.7999878
Thalidomide	248.8	280.4006958
Cabozantinib	412.3	401.675293
Sorafenib	290.3	312.9441528
Sunitinib	299.8	320.2272034
Axitinib	358.3	315.0227661
Lenvatinib	333.1	321.8497925
Erlotinib	288.6	297.0558777
Neratinib	411.6	417.0478516
Ifosfamide	157.1	200.4196777
Cytarabine	283.8	253.5572205
Docetaxel	498.4	504.6493835
Paclitaxel	532.6	542.1871948
Valrubicin	478.6	472.5236816
Mitomycin C	305.6	322.8450623
Erdafitinib	354.4	349.6944275
Gemcitabine	245.7	269.0063171

**Table 28:** Performance metrics.

MSE	MAE	RMSE	$R^2$
589.18	19.98	24.27	0.93

**Fig. 15:**
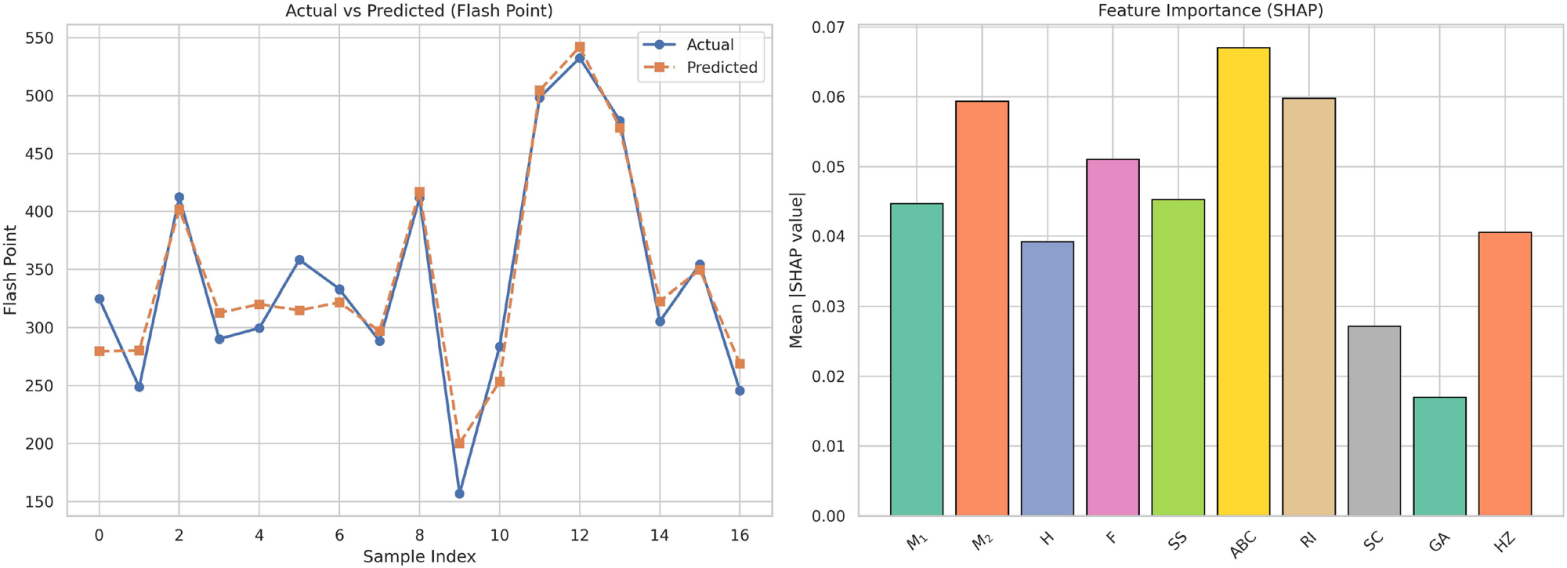
Graphical representation of Flash Point.

The findings in Table 27 illustrate tight concordance of predicted and actual flash points of chosen medications. Although slight variations occur in particular examples, e.g., Lenalidomide and Cytarabine, overall performance metrics in Table 28 confirm the predictive power of the model. A high $R^2$ measure value (0.93) ensures that most of the variance in flash point values is explained by the model. Low values of MAE (19.98), RMSE (24.27), and an understandable value of MSE (589.18) affirm generalizability and robustness of the regression model

Figure 15 is used to qualitatively measure the predictive power of the model. The line plot of predicted versus actual flash points illustrates that predicted values closely track actual data, further supporting the robustness of the model for various compounds. In addition, the SHAP feature importance plot allows identification of the most significant descriptors used for flash-point prediction. Indeed, descriptors like ABC and H feature higher SHAP values, reflecting that these descriptors play an important role in determining flash-point behavior of the drugs. Such an understanding can assist in the rational design of drug compounds possessing desirable thermophysical characteristics.

**Table 29:** Actual vs predicted polar surface area of selected drugs.

Drug	Actual Polar Surface Area	Predicted Polar Surface Area
Lenalidomide	93	98.64737701
Thalidomide	87	98.75508881
Cabozantinib	99	115.8902969
Sorafenib	92	93.38889313
Sunitinib	77	94.1081543
Axitinib	96	84.89568329
Lenvatinib	116	99.37735748
Erlotinib	75	79.48949432
Neratinib	112	120.8940735
Ifosfamide	51	65.32022858
Cytarabine	112	91.38275909
Docetaxel	224	231.3431091
Paclitaxel	222	246.5342712
Valrubicin	245	198.7397461
Mitomycin C	147	146.6862488
Erdafitinib	77	81.61560059
Gemcitabine	108	113.4434052

**Table 30:** Performance metrics.

MSE	MAE	RMSE	$R^2$
277.99	12.78	16.67	0.91

**Fig. 16:**
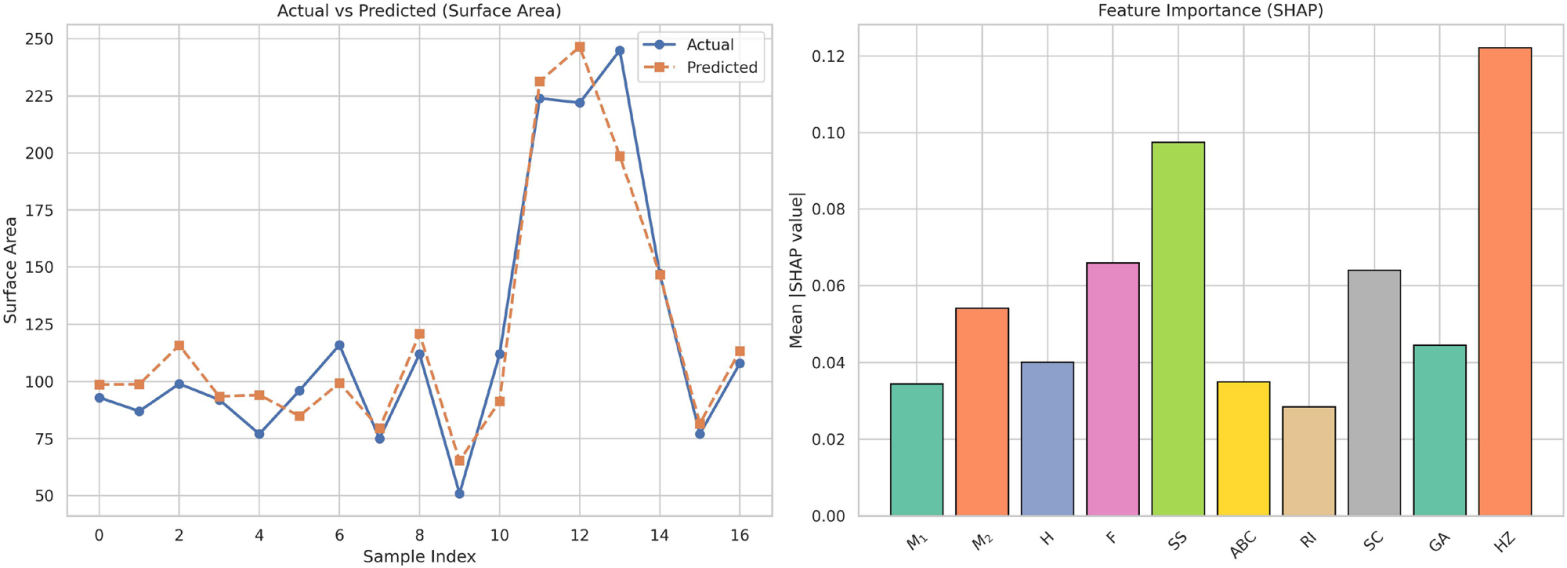
Graphical representation of Polar Surface Area.

Table 29 shows the comparison of actual vs predicted polar surface areas of certain drugs. The model is quite accurate in most compounds, and slight deviation is noticed in only a few like Cytarabine and Valrubicin. Performance metrics in Table 30 reinforce the strong predictive power of the model, as indicated by high $R^2$ value of 0.91. The model also gives relatively low MAE (12.78), RMSE (16.67), and MSE (277.99) values, portraying it as a reliable one to predict polar surface area.

Figure 16 illustrates the graphical comparison between actual and predicted polar surface areas and the corresponding SHAP-based feature importance. The trend alignment between actual and predicted values in the line plot validates the model’s ability to generalize across diverse molecular structures. The SHAP plot highlights the relative influence of molecular descriptors, with the descriptor HZ showing the highest importance, followed by SS and H. These insights suggest that these descriptors play a significant role in determining polar surface area, providing valuable direction for future feature selection and model refinement efforts.

**Table 31:** Actual vs predicted molar volume of selected drugs.

Drug	Actual Molar Volume	Predicted Molar Volume
Lenalidomide	177.5	164.1250763
Thalidomide	161	164.0933075
Cabozantinib	359	377.567688
Sorafenib	319.5	322.9491272
Sunitinib	324.1	286.0852966
Axitinib	284.8	284.7854919
Lenvatinib	280.6	297.2623596
Erlotinib	315.4	305.878479
Neratinib	416.8	399.3868408
Ifosfamide	195.7	148.6885376
Cytarabine	128.4	153.9259033
Docetaxel	585.7	565.6398926
Paclitaxel	610.6	619.2505493
Valrubicin	469.8	482.9135437
Mitomycin C	213.7	202.4745178
Erdafitinib	389.7	373.9685364
Gemcitabine	142.3	152.5334015

**Table 32:** Performance metrics.

MSE	MAE	RMSE	$R^2$
391.22	15.98	19.77	0.98

**Fig. 17:**
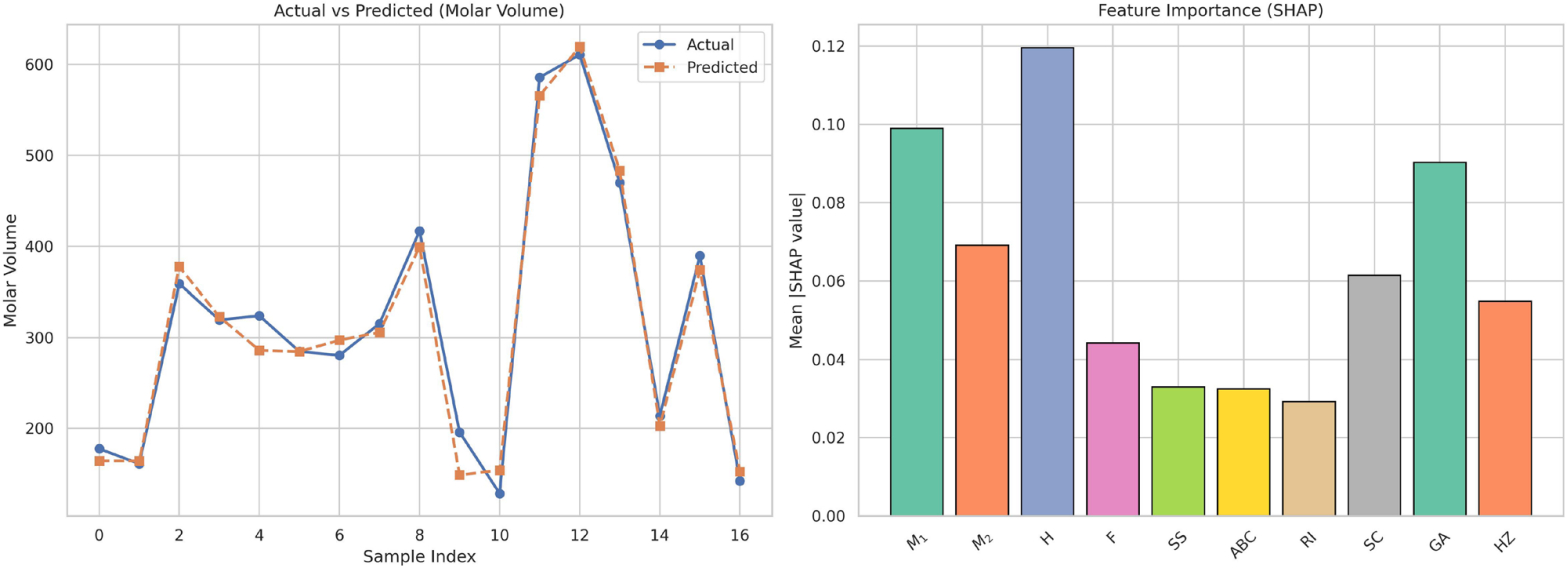
Graphical representation of Molar Volume.

Table 31 presents the actual vs. predicted molar volumes of chosen molecules. The predictions match closely to real values for most compounds, showing slight underpredictions in compounds like Sunitinib and Ifosfamide. The efficiency of the model is quantitatively evidenced by Table 32, which provides an exceptionally high $R^2$ value of 0.98. The MAE (15.98), RMSE (19.77), and MSE (391.22) are also relatively low, suggesting that the model is highly accurate and consistent in estimating the molar volume of chemically diverse compounds. Figure 17 also gives visual validation of the working of the model. The line plot of predicted vs actual molar volumes shows close correspondence, where the predicted values closely follow the actual trend, attesting to the fitness of the regression model. The SHAP feature importance plot also discloses the contribution of molecular descriptors, where H and $M_1$ turn out to be the most significant features, followed by HZ and GA. The descriptors seem to be crucial to embodying structural or physicochemical features of relevance to molar volume, and thus provide insight into optimizing feature sets in predictive modeling endeavors.

**Table 33:** Actual vs predicted polarizability of selected drugs.

Drug	Actual Polarizability	Predicted Polarizability
Lenalidomide	26.3	24.58438492
Thalidomide	25.9	24.48139381
Cabozantinib	54.3	54.7753067
Sorafenib	44.8	44.9601059
Sunitinib	44.6	43.56602478
Axitinib	45	44.12747955
Lenvatinib	44.4	44.91093063
Erlotinib	43.6	43.86928558
Neratinib	61.5	61.14069748
Ifosfamide	23	19.85934639
Cytarabine	20.9	22.25996971
Docetaxel	81.4	79.37337494
Paclitaxel	86.9	86.46870422
Valrubicin	65.3	68.3669281
Mitomycin C	32	31.10996819
Erdafitinib	51.4	49.82715607
Gemcitabine	20.6	22.71489334

**Table 34:** Performance metrics.

MSE	MAE	RMSE	$R^2$
2.39	1.25	1.54	0.99

**Fig. 18:**
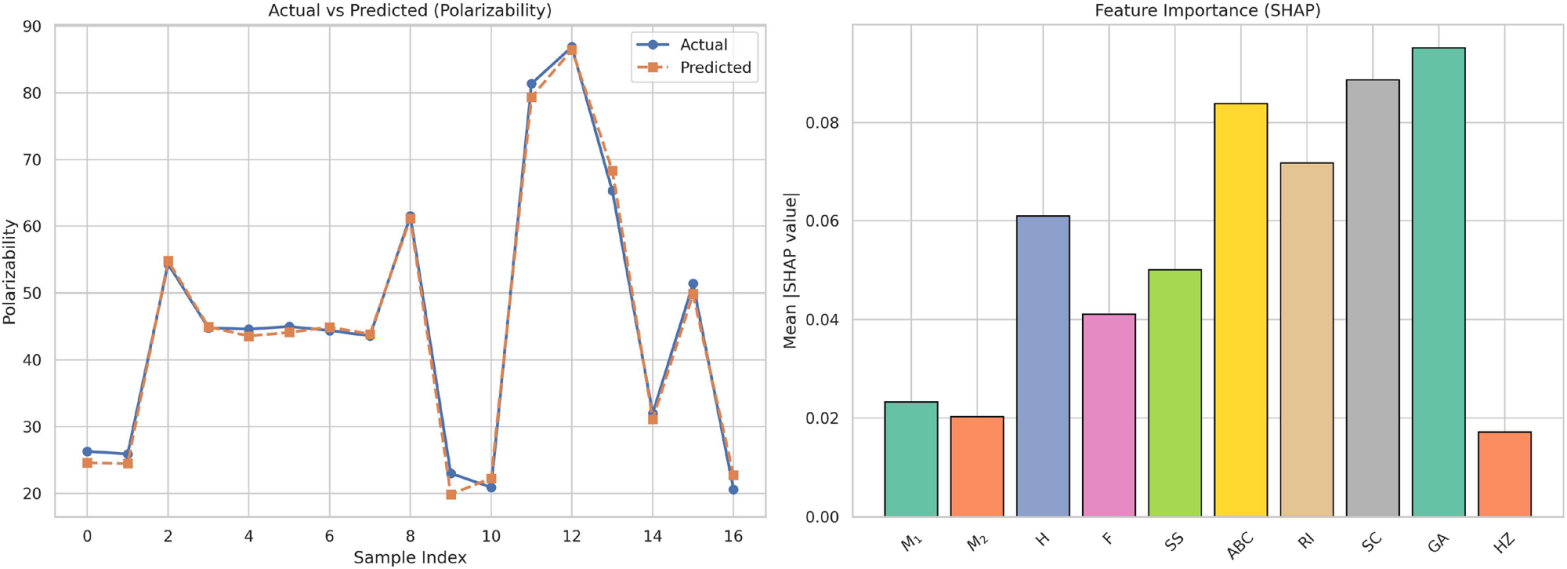
Graphical representation of polarizability.

Table 33 shows that there is close correspondence between predicted and actual polarizability values of a set of drugs. The fact that highly varying drugs like Cabozantinib, Paclitaxel, and Neratinib have negligible errors in prediction is a reflection of strong generalizability of the prediction model to compounds that vary greatly in terms of polarizability range. The metrics of performance tabulated in Table 34 also affirm this fact, as there is low deviation shown by them (MSE = 2.39, MAE = 1.25, RMSE = 1.54) and also there is maximum correspondence of predicted values with actual values as evidenced by high value of $R^2 = 0.99$.

Figure 18 provides visual insight into the predictive power of the model. The line graph of predicted vs actual polarizability shows that there is a correlation of trend, which is corroborated by the quantitative measures presented in the tables. Additionally, the SHAP (SHapley Additive exPlanations) feature importance plot gives insight into what individual descriptors contribute to the output of the model. Clearly, features like GA, ABC, and RI contribute most to polarizability prediction, while features like HZ and HM contribute relatively less to it. This graphical analysis not only confirms predictions made by the model but also gives interpretability, leading to further feature optimization and feature selection

## Conclusion

The current work shows that artificial neural networks and polynomial regression models in combination with graph-theoretic descriptors can be used to predict the polarizability of bladder cancer medicines effectively. Topological indices, based on degree, were able to develop both linear and cubic regression models, of which cubic provided better fitting of non-linear correlations. The ANN trained on an identical set of descriptors performed decidedly better than regression models as predictors, recording a remarkable $R^2$ of 0.99 and negligible prediction errors. The SHAP analysis also facilitated the black-box ANN model’s interpretability by identifying descriptors with the strongest influences. This hybrid method not only confirms the reliability of topological indices in QSPR/QSAR modeling but also encourages the employment of ANN as an effective predictive method in cheminformatics. The framework is extendible to other disease categories and molecular libraries, which has significant implications for computational drug design and high-throughput screening.

## Data Availability

The datasets used and/or analysed during the current study available from the corresponding author on reasonable request.
